# Implementing smoke-free policies in low- and middle-income countries: A brief review and research agenda

**DOI:** 10.18332/tid/110007

**Published:** 2019-08-05

**Authors:** M. Justin Byron, Joanna E. Cohen, Shannon Frattaroli, Joel Gittelsohn, Jeffrey M. Drope, David H. Jernigan

**Affiliations:** 1 Department of Family Medicine, School of Medicine, University of North Carolina, Chapel Hill, United States; 2 Department of Health Behavior, Gillings School of Global Public Health, University of North Carolina, Chapel Hill, United States; 3 Lineberger Comprehensive Cancer Center, University of North Carolina, Chapel Hill, United States; 4 Department of Health, Behavior and Society, Johns Hopkins Bloomberg School of Public Health, Baltimore, United States; 5 Department of Health Policy and Management, Johns Hopkins Bloomberg School of Public Health, Baltimore, United States; 6 Department of International Health, Johns Hopkins Bloomberg School of Public Health, Baltimore, United States; 7 American Cancer Society, Atlanta, United States; 8 Department of Political Science, Marquette University, Milwaukee, United States; 9 Department of Health Law, Policy & Management, Boston University School of Public Health, Boston, United States

**Keywords:** smoke-free policy, developing countries, review, tobacco smoke pollution, tobacco use

## Abstract

**INTRODUCTION:**

Some low- and middle-income countries (LMICs) struggle to implement smoke-free policies. We sought to review the academic and gray literature, and propose a research agenda to improve implementation of smoke-free policies and make them more effective in LMICs.

**METHODS:**

We reviewed 10 databases for variations of (‘implementation’ /‘enforcement’ /‘compliance’) and (‘smoke-free’ /‘ban’ /‘restriction’) and (‘tobacco’ /‘smoking’). We also reviewed cited sources and the gray literature including non-governmental organization reports.

We included articles that described problems that arose, attempted solutions, lessons learned, and research questions posed regarding smoke-free policy implementation in LMICs. We excluded studies of high-income countries, institution-level implementation, voluntary smoke-free policies, smoke-free homes, and outdoor smoke-free policies.

**RESULTS:**

The academic literature review led to 4931 unique articles, reduced to 1541 after title screening, 331 after abstract screening, and 101 after full-text review. The citation and gray literature review led to an additional 179 publications of which 67 met the inclusion criteria. In total we retained 168 sources. We conducted a narrative review and synthesis of the literature, extracting key themes and noting research gaps.

**CONCLUSIONS:**

We find that progress is urgently needed in five categories: identifying the critical lessons learned for effective implementation, evaluating different enforcement approaches, learning how to rejuvenate stalled smoke-free policies, learning how to increase ground-level will to enforce policies, and developing a conceptual framework that explains implementation. Investigation into these topics can improve implementation of smoke-free policies in LMICs.

## INTRODUCTION

Globally, secondhand smoke leads to 1.220 million deaths per year, of which 1.091 million are in low- and middle-income countries^1^. Among adults, secondhand smoke causes immediate cardiovascular effects, coronary heart disease, lung cancer, and potentially an array of other cancers^2^. In children, secondhand smoke causes sudden infant death syndrome, acute respiratory infections, and additional health problems^2^. The only way to fully protect non-smokers from the effects of smoke is to make spaces smoke-free^2^. Effective smoke-free policies banning tobacco smoking in public places and workplaces reduce exposure to smoke^3,4^, mitigate harmful health outcomes^5,6^, reduce smoking prevalence^7,8^, denormalize tobacco use^9^, and potentially discourage youth smoking initiation^10,11^. While smoke-free policies (laws, by-laws, ordinances, regulations etc.) are becoming more common, more than 80% of the world’s population is not yet protected by these policies^12^. The World Health Organization (WHO) recommends that all countries implement comprehensive smoke-free policies, defined as smoke-free policies with no exemptions for particular venue types or allowances for designated smoking areas^2,13,14^. There are now 181 countries that are parties to the WHO Framework Convention on Tobacco Control (FCTC), an international treaty of evidence-based public health measures responding to the tobacco epidemic^15^. The FCTC requires parties to implement smoke-free policies^16^ within 5 years of the FCTC taking force^17^, although accountability is limited^18^. Most of the first smoke-free policies were passed in high-income countries (HICs), where they have generally, but not always^19^, been popular with the public and achieved and maintained high compliance^13^. Starting with Uruguay in 2006, some low- and middle-income countries (LMICs) are implementing comprehensive smoke-free policies. This is important, as the burden of the tobacco epidemic and resulting deaths have shifted to LMICs^20^, with 77% of all smoking-related deaths and 89% of secondhand smoke related-deaths occurring in LMICs^1^. As of 2016, 92 of the 138 LMICs have passed a smoke-free policy, but only 39 have comprehensive policies^14^. WHO has called for parties to the FCTC to conduct relevant research, share best practices, and assist LMICs with implementing effective tobacco control measures^21^. We sought to review the academic and gray literature and propose a research agenda to improve implementation of smoke-free policies and make them more effective in LMICs.

## METHODS

**Figure 1:**
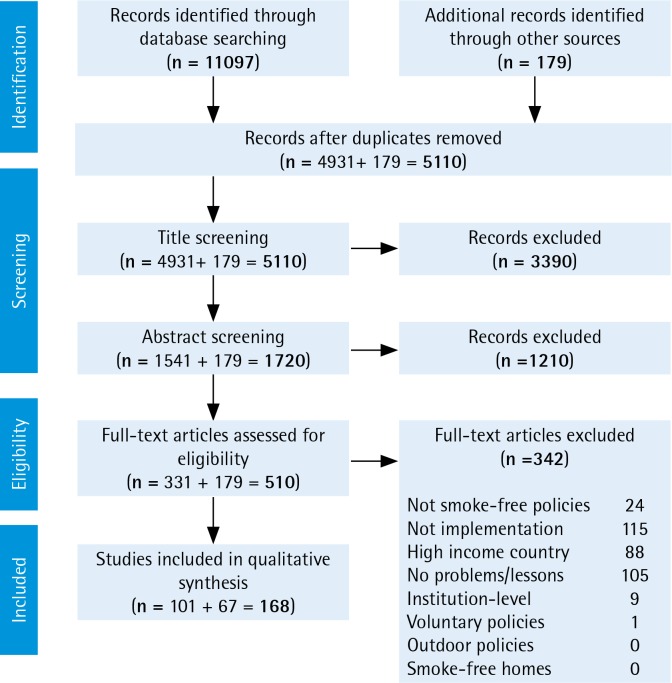
PRISMA diagram of document review

We reviewed published academic and gray literature, and compiled lessons learned from experience working on smoke-free implementation in LMICs. We began with a comprehensive review using 10 health-related databases: PubMed, Embase, Cochrane Reviews, CINAHL, Global Health (OVID), PAIS International, PsycINFO, Scopus, Sociological Abstracts, and Web of Science, and searched for articles published through 18 January 2017. We searched for combinations and suffix variations of (‘implementation’ or ‘enforcement’ or ‘compliance’) and (‘smoke-free’ or ‘ban’ or ‘restriction’) and (‘tobacco’ or ‘smoking’). Full search strings are given in Supplementary File 1. To meet the inclusion criteria, articles needed to address at least one of the following: problems that have arisen, attempted solutions, lessons learned, or research questions posed regarding smoke-free policy implementation in LMICs. Articles were excluded if they only covered research in high-income countries, institution-level implementation, voluntary smoke-free policies, smoke-free homes, or outdoor smoke-free policies. We limited the search to the implementation of smoke-free policies at the city, regional, or national level. The search yielded 11097 total articles, of which 4931 were unique (Figure 1). The count reduced to 1541 after title screening, 331 after abstract screening, and 101 after full-text review. For 20 articles in which full text was not available in English, we reviewed the English abstracts. As a second major data source, we reviewed relevant citations from the found articles and extracted reports from WHO and tobacco control non-governmental organizations (NGOs). Of these additional 179 publications, 67 met the inclusion criteria used in the academic literature review, bringing the total number of search-based sources to 168. Most of the included articles were selected for meeting the criteria of discussing problems faced or lessons learned. Additionally, we incorporated relevant findings from our own work on smoke-free policies in South-East Asia^22,23^, Africa^24,25^, and globally^26-30^. We focus on post-legislation implementation — which we define as putting a smoke-free policy into practice and enforcing it to achieve the public’s compliance with the policy. After the articles were selected, we conducted a narrative review and synthesis of the literature^31^. First, we reviewed each source and catalogued key findings and indications of research needs. We then conducted a thematic content analysis in which we extracted recurrent themes from the data and noted research gaps^32^. Finally, the research team reviewed summary documents and discussed the data organization and presentation.

## RESULTS and DISCUSSION

The review revealed that many findings about smoke-free policies in HICs carry over to LMICs. As in HICs, successful smoke-free policies in LMICs reduce smoke exposure^33-35^ and resulting health harms^34,36-39^, increase quit intentions^40,41^, and potentially decrease youth smoking initiation^42^. Also as in HICs, policies that do not allow for designated smoking areas are easier to implement^43-45^ and more effective in reducing smoke exposure^28,35,46,47^. There is generally a high level of public support for smoke-free policies in LMICs^34,48-60^ and public support increases after implementation^33,48,61-63^. Research in LMICs also finds that there is no negative economic impact of smoke-free policies on the hospitality industry^64,65^. While these findings bode well for smoke-free policies that are successfully implemented, the actual implementation of smoke-free policies in LMICs has been mixed^14,24,63,66^.

The review found five priority gaps in knowledge. First, based on the experiences of cities, regions, and countries worldwide, there is now perhaps an unwieldy number of recommendations, guidelines, and lessons learned for implementing smoke-free policies^13,27,37,64,67-86^. Second, multiple approaches of enforcement have been used, but they have not been directly compared to see which is most effective^13,17,67^. Third, there was a common report of problems in enforcement, i.e. the ground-level implementers (inspectors, police, venue managers etc.) often do not enforce the policy^14,63,66^. Fourth, there are a number of countries where smoke-free policies exist on paper but not in practice, and they are in need of rejuvenation^14,24,63,66^. Fifth, the literature presented a notable gap in the conceptual framework with which to organize, troubleshoot, and advance smoke-free policies^87-93^. A table of the sources is provided in Supplementary File 2.

Here, we present the review findings and an agenda of the key areas where research can make a difference in advancing implementation of smoke-free policies. Each section begins with the current evidence, an estimation of weight of the evidence, and the research gaps for the topic. We then provide recommendations for the research needed. We present the five areas of findings and needed research in approximate order of potential impact.

### Identifying the critical lessons learned for effective implementation

In addition to the many case studies documenting experiences of LMIC jurisdictions implementing smoke-free policies^27,37,64,71-84^, WHO and various NGOs have compiled lessons learned from numerous countries^13,67-69,85,86^. These guides suggest the following attributes may be most important to successful implementation of smoke-free policies in HICs and LMICs:

- Strong political leadership;
- Legislation that is simple, clear, enforceable, and comprehensive;
- Thoughtful planning and adequate resources for implementation and enforcement;
- Preparing for and countering tobacco industry opposition;
- Involvement of civil society in planning and implementation;
- Public education and outreach;
- Education and consultation with stakeholders;
- Monitoring and evaluation^13,67,68,85,86^.

Additionally, these documents describe in detail many potentially important practices that complement the above identified attributes. A sampling of these additional practices include engaging in early interagency planning, securing adequate financial resources, assigning enforcement responsibility to the most effective agency, requiring removal of ashtrays from smoke-free areas, framing education messages around the health benefits of the policy for workers, focusing enforcement on venue managers rather than individual violators, and providing the public with a way to report violations^17,67-69,85,94^. The weight of the evidence in terms of there being a large body of recommendations is strong; the gap remains in refining these lists, since most LMICs have limited resources with which to implement smoke-free policies^25,95^.

The resulting area of research needed is in identifying those recommendations most critical for success. With this information, officials in LMICs can create an implementation plan compatible with their resources that covers the most essential recommendations. Interviews and open-ended surveys with government officials and tobacco control NGOs in LMIC jurisdictions where smoke-free policies are working well would be good approaches for learning these essential best practices. Researchers could also contribute to creating and testing basic low-cost instruments to assess the social environment and logistical resources before a smoke-free policy is passed. Such evaluations can examine public awareness of the proposed policy, the public’s compliance with similar policies (e.g. littering and other public conduct policies), experience and realistic capabilities of potential enforcement staff (e.g. police, health inspectors), and other key data points. With this needs-assessment, strategies can be organized to best address the known weak points and allocate financial and human resources accordingly for the particular country.

### Evaluating different enforcement approaches

Jurisdictions across LMICs have tried different approaches to enforce smoke-free policies. For example, the early stages of enforcement may be especially important in setting the tone for the policy^17,67^. There are differing opinions as to the effectiveness of ‘soft enforcement’, an approach of phasing-in enforcement starting with reminders rather than fines^13,17,67^. On one hand, a ‘grace period’ is a way of educating people unaware of the policy and reasonably addressing violations due to ignorance^13^. On the other hand, strict enforcement, providing fines from day one, shows that a government is serious about the policy, and will enforce it^67^. A compromise approach is to use strict enforcement but allow businesses one formal warning before fines are issued^67^. Another set of questions is around the types of enforcement strategies to use — proactive versus reactive, random versus focused, and overt versus covert^67^. Finally, an important component to smoke-free policy enforcement is determining the allocation of enforcement resources and the size of fines for violations^96^. The weight of the literature on the need for optimizing enforcement is moderate; the research gap is in rigorously comparing different approaches and determining the most effective methods.

There is a need for empirical evidence on how to most effectively enforce smoke-free policies. Research is needed on how long a grace period (if any) is optimal and whether there are contextual differences across LMICs that suggest different approaches for different jurisdictions. The different enforcement strategies should be studied and compared for effectiveness. There is also a need for research on the most cost-effective ways of enforcing smoke-free policies. Should equal effort be put into training city officers, educating venue managers, and communicating to the public about their role in enforcement? What size fines work best in getting attention while still being realistic and fair to people of all income levels? Answering these questions could make implementation more efficient and effective. Research approaches can include analysis of what has been done across various jurisdictions to date. Additionally, researchers can test new technologies, such as mobile phone apps, for reporting violations, that may support enforcement in relatively inexpensive ways^97,98^.

### Learning how to rejuvenate stalled smoke-free policies

There are numerous cases where smoke-free policies have been enacted but are not achieving high compliance^14,24,63,66^. In some cases, no implementation plan was made after a policy was passed. In other cases, implementation was weak or compliance faded over time. Civil society and government organizations have used various tactics for reinvigorating smoke-free policies. In India, joint government–NGO communication campaigns, legal actions seeking information from enforcement officials, and youth-led education efforts aided better compliance^72^. In Mexico City, a clearer, comprehensive smoke-free policy was used to replace a more cumbersome policy that allowed venues to have designated smoking areas^76^. In the Philippines, advocates created a task force of people in government service that worked cooperatively with the business sector, medical groups, and city government to raise awareness and compliance^68^. The weight of the literature on the problem of stalled smoke-free policies is moderate; the research gap is learning approaches and tools to reinvigorate these policies.

An important line of research is the development of a set of proven approaches or tools for improving compliance in places where implementation has stalled. Some areas to consider are: where the weak points of the particular implementation effort are, how to assess the appropriateness of the enforcement agency, how to re-establish political will to enforce policies if they fade, when to launch a new communication campaign (and what the focus and target audience of the campaign should be)^84^, and how to involve new organizations or constituencies in the enforcement effort. Approaches that have successfully been used in the past^67,68,76^ would benefit from replication, refinement, and consolidation into a resource for LMICs.

### Learning how to increase ground-level will to enforce policies

In some LMICs, there is a lack of motivation to enforce smoke-free policies at the ground level^24,25,66,82,99-101^. Enforcement of smoke-free policies typically involves both enforcement officers and venue managers. Enforcement officers may not feel they have time or may think that public smoking is not a problem that warrants their attention. Some enforcement officers have said they have more important crimes to address^101^. Others may lack the will to enforce a policy because of lack of confidence that the government will back them if they are challenged^25^. In other cases, it is the regional health departments that are not sufficiently supportive^66^. In restaurants and retail locations, venue managers report having conflicts about enforcing a policy among their customers, on whom their livelihood depends^27,99^. Some managers have said they would prefer if a government agency did the enforcement rather than asking them to do it^99^. The weight of the evidence on the problem of ground-level enforcement is moderate; the research gap is in learning how to address this problem.

Research is needed on what actions can be taken to increase the will to enforce a smoke-free policy. Possible solutions may involve actions by civil society to show public support for smoke-free venues or using public shaming or praise to motivate action. Researchers can also examine how various jurisdictions have successfully addressed this problem and then replicating these approaches. Existing reports, of lessons learned, detail some successful creative efforts by members of civil society to hold governments accountable and increase ground-level enforcement^24,68^, but there has been little systematic work to identify if the necessary tactics vary by cultural context or legal structures.

### Developing a conceptual framework that explains implementation

The review found little discussion of the behavioral science or policy theory involved in implementing smoke-free policies. In 1991, Pederson et al.^87^ created a preliminary model hypothesizing that compliance with smoke-free policies is a factor of environmental support, personality, and attitudes. More recently, a qualitative study in Israel used the behavioral ecological model to explore contingencies of reinforcement around non-compliance with a smoke-free policy in pubs and bars^88^. Work has also been conducted in Albania, Bulgaria and Greece to look into predictors of compliance, as well as non-smokers’ assertiveness to confront smokers^89-92^. The literature shows a lack of a general conceptual framework that explains how implementation of smoke-free laws works. The weight of evidence for the need for a conceptual framework is moderate to low; the gap that remains is in integrating and updating existing work into a cohesive framework.

An accurate conceptual framework for how smoke-free policies work would help guide efficient implementation of new smoke-free policies and troubleshoot existing implementation efforts. This framework could be designed to explain the social and psychological processes involved in changing a smoking social norm to a non-smoking one. Such a framework could inform communications messaging, enforcement, and advocacy approaches. Sources for framework development could come from a variety of relevant fields including behavioral science, communications, and criminal justice. Existing theoretical work can be incorporated into a new conceptual framework that considers:

- How do social norms change and legal enforcement intersect in creating a successful smoke-free policy, and what factors matter most? What is the process by which a policy becomes ‘self-enforcing’?
- Do differences such as whether a society is collectivist/individualist or the type of governance matter in how a smoke-free policy is best implemented? For example, poor compliance with a smoke-free policy in China was explained by a strong cultural desire to keep harmony and avoid disputes with others, leaving people unwilling to confront smokers^36^.
- Are different enforcement approaches needed in different venue types? Schools, public transportation, government offices, restaurants, private workplaces, worship venues etc. have different social and power dynamics that may influence enforcement. For example, business managers can punish subordinates who smoke, but restaurant managers may not want to offend customers. Understanding these dynamics could lead to more effective enforcement methods.
- How do changes in the environment (e.g. advertising bans, counter-marketing campaigns) affect public compliance with smoke-free policies?^102-104^

A conceptual framework would be a valuable way to frame, organize, and improve implementation of smoke-free policies.

## CONCLUSIONS

We believe the research in the avenues presented can assist in strengthening and streamlining implementation of smoke-free policies in LMICs and thus improve public health. We propose research in five major categories: identifying the critical lessons learned for effective implementation, evaluating different enforcement approaches, learning how to rejuvenate stalled smoke-free policies, learning how to increase ground-level will to enforce policies, and developing a conceptual framework that explains implementation. Research in these topics is both feasible and potentially powerful in advancing successful implementation of smoke-free policies in LMICs to protect public health.

## CONFLICTS OF INTEREST

The authors have completed and submitted the ICMJE Form for Disclosure of Potential Conflicts of Interest and none was reported.
