# Supplementary material for: Implementing smoke-free policies in low- and middle-income countries: A brief review and research agenda
Source: Tob Induc Dis. 2019 Aug 5;17:60. doi: 10.18332/tid/110007 (PMC6770618; doi:10.18332/tid/110007)
Supplement: Supplementary file 1 [file TID-17-60-s1.pdf]

Supplementary File 1. Search Strings

PubMed

(implement[tw] OR implementation[tw] OR implementing[tw] OR implemented[tw] OR enforce[tw] OR enforcement[tw] OR enforcing[tw] OR enforced[tw] OR comply[tw] OR compliance[tw] OR complying[tw] OR complied[tw] OR "guideline adherence"[MeSH Terms] OR "policy enforcement"[MeSH Terms]) AND (smoke-free[tw] OR smokefree[tw] OR "smoke free"[tw] OR ban[tw] OR bans[tw] OR banning[tw] OR banned[tw] OR restrict[tw] OR restriction[tw] OR restrictions[tw] OR restricting[tw] OR restricted[tw]) AND (("tobacco"[MeSH Terms] OR "tobacco products"[MeSH Terms]) OR tobacco[tw] OR "tobacco products"[MeSH Terms] OR "smoking"[MeSH Terms] OR smoking[tw] OR "smoke"[MeSH Terms] OR smoke[tw])

Embase

(implement OR implementation OR implementing OR implemented OR enforce OR enforcement OR enforcing OR enforced OR comply OR compliance OR complying OR complied OR "guideline adherence" OR “policy enforcement”) AND (smoke-free OR smokefree OR "smoke free" OR ban OR bans OR banning OR banned OR restrict OR restriction OR restrictions OR restricting OR restricted) AND (tobacco OR tobacco OR "tobacco products" OR smoking OR smoking OR smoke OR smoke)

Global Health, CINAHL, PsychINFO

(implement OR implementation OR implementing OR implemented OR enforce OR enforcement OR enforcing OR enforced OR comply OR compliance OR complying OR complied) AND (smoke-free OR smokefree OR ban OR bans OR banning OR banned OR restrict OR restriction OR restrictions OR restricting OR restricted) AND (tobacco OR tobacco OR smoking OR smoke OR smoke)

Cochrane

(smoke-free OR smokefree OR "smoke free" OR ban OR bans OR banning OR banned OR restrict OR restriction OR restrictions OR restricting OR restricted) AND (tobacco OR tobacco OR "tobacco products" OR smoking OR smoking OR smoke OR smoke)

Note: because few articles appeared when including the "implementation" terms, a broader search, above, was used.

SCOPUS

TITLE-ABS-KEY((implement OR implementation OR implementing OR implemented OR enforce OR enforcement OR enforcing OR enforced OR comply OR compliance OR complying OR complied OR "guideline adherence" OR "policy enforcement") AND (smoke-free OR smokefree OR "smoke free" OR ban OR bans OR banning OR banned OR restrict OR restriction OR restrictions OR restricting OR restricted) AND (tobacco OR tobacco OR "tobacco products" OR smoking OR smoking OR smoke OR smoke)) AND (EXCLUDE(SUBJAREA,"BIOC") OR EXCLUDE(SUBJAREA,"PHAR")) AND (EXCLUDE(SUBJAREA,"COMP") OR EXCLUDE(SUBJAREA,"COMP"))

PAIS+ Sociological Abstracts

(implement OR implementation OR implementing OR implemented OR enforce OR enforcement OR enforcing OR enforced OR comply OR compliance OR complying OR complied OR "guideline adherence" OR “policy enforcement”) AND (smoke-free OR smokefree OR "smoke free" OR ban OR bans OR banning OR banned OR restrict OR restriction OR restrictions OR restricting OR restricted) AND (tobacco OR tobacco OR "tobacco products" OR smoking OR smoking OR smoke OR smoke)

Web of Science

TS=((implement OR implementation OR implementing OR implemented OR enforce OR enforcement OR enforcing OR enforced OR comply OR compliance OR complying OR complied OR "guideline adherence" OR “policy enforcement”) AND (smoke-free OR smokefree OR "smoke free" OR ban OR bans OR banning OR banned OR restrict OR restriction OR restrictions OR restricting OR restricted) AND (tobacco OR tobacco OR "tobacco products" OR smoking OR smoking OR smoke OR smoke)

© 2019 Byron M.J.
