# Supplementary material for: Implementing smoke-free policies in low- and middle-income countries: A brief review and research agenda
Source: Tob Induc Dis. 2019 Aug 5;17:60. doi: 10.18332/tid/110007 (PMC6770618; doi:10.18332/tid/110007)
Supplement: Supplementary file 2 [file TID-17-60-s2.pdf]

First author	Year	Country	Source type	Abstract or summary	Citation
Abdullah et al.	2010	China	Article	Background: In 2005 China ratified the WHO Framework Convention on Tobacco Control (FCTC) and committed to implement tobacco control legislation and policies. Wide variation in smoking prevalence between men and women in China and the high exposure of women to secondhand smoke suggest that each component of the FCTC should be analyzed from a gender perspective. This study describes women's attitudes toward and predictors of support for four key FCTC measures in China. Methods: Cross-sectional data were collected from 1,408 women in two urban cities on demographics, smoking behavior, and attitudes toward key tobacco control measures. Results: Seventy percent of the study women (n=1,408) were exposed to secondhand smoke at home, work, or other public places. Support for the four FCTC measures of interest was as follows: 92.5% supported banning smoking in public places, 79.2% supported increasing the cigarette tax, 92% supported stronger health warnings on cigarette packages, and 87.1% favored banning tobacco advertising. The predictors for supporting each of these measures included socioeconomic, attitudinal, and behavioral factors. Conclusion: Urban Chinese women appear to support implementation of key WHO FCTC measures. Predictors of women's attitudes toward the key FCTC measures varied. The formulation process resulting from the tobacco control policy should consider these women-specific predictors in order to facilitate successful implementation of FCTC.	A.S. Abdullah, T. Yang, J. Beard. Predictors of women's attitudes toward world health organization framework convention on tobacco control policies in urban China. J Womens Health. 2010;19(5):903-909. doi: 10.1089/jwh.2009.1613
Abe et al.	2016	Brazil	Article	Background: Smoking restriction laws have spread worldwide during the last decade. Previous studies have shown a decline in the community rates of myocardial infarction after enactment of these laws. However, data are scarce about the Latin American population. In the first phase of this study, we reported the successful implementation of the law in Sao Paulo city, with a decrease in carbon monoxide rates in hospitality venues. Objective: To evaluate whether the 2009 implementation of a comprehensive smoking ban law in Sao Paulo city was associated with a reduction in rates of mortality and hospital admissions for myocardial infarction. Methods: We performed a time-series study of monthly rates of mortality and hospital admissions for acute myocardial infarction from January 2005 to December 2010. The data were derived from DATASUS, the primary public health information system available in Brazil and from Mortality Information System (SIM). Adjustments and analyses were performed using the Autoregressive Integrated Moving Average with exogenous variables (ARIMAX) method modelled by environmental variables and atmospheric pollutants to evaluate the effect of smoking ban law in mortality and hospital admission rate. We also used Interrupted Time Series Analysis (ITSA) to make a comparison between the period pre and post smoking ban law. Results: We observed a reduction in mortality rate (-11.9% in the first 17 months after the law) and in hospital admission rate (-5.4% in the first 3 months after the law) for myocardial infarction after the implementation of the smoking ban law. Conclusions: Hospital admissions and mortality rate for myocardial infarction were reduced in the first months after the comprehensive smoking ban law was implemented.	T. M. Abe, J. Scholz, E. de Masi, M. R. Nobre and R. K. Filho. Decrease in mortality rate and hospital admissions for acute myocardial infarction after the enactment of the smoking ban law in Sao Paulo city, Brazil. Tobacco control. 2016;():.
Abidin et al.	2014	Malaysia	Article	Objective: To identify the relationship between knowledge, attitude and perception regarding environmental tobacco smoke (ETS) and smoking among Malaysian adolescents living in states with complete or partial smoke-free legislation (SFL). Methods: A total of 898 respondents aged 13-14 years were randomly selected from 21 secondary schools. The Malay version of the modified Global Youth Tobacco Survey questionnaire was used. Hierarchical logistic regression was performed in examining predictors of smoking attempt among adolescents. Results: Participants exposed to ETS >5 h/day were more likely to have smoked compared to those exposed to ETS <1 h/day (OR 4.1, 95%CI 1.03-16.0). Living in a state with partial SFL was associated with a two-fold increase in self-reported smoking attempts (OR 1.95, 95%CI 1.10-3.43) compared to living in a state with complete SFL. Negative attitudes and perceptions towards smoking and ETS exposure were linked to lower smoking attempts in states with complete SFL. Conclusions: Adolescents with limited ETS exposure who lived in a state with complete SFL were less likely to attempt smoking compared to those exposed more regularly to ETS and living in a state with partial SFL. Preventing adolescents from becoming smokers is the key to reducing national prevalence rates in smoking. There is a need to implement comprehensive smoke-free legislation nationally across Malaysia.	N. Z. Abidin. Knowledge, attitude and perception of second-hand smoke and factors promoting smoking in Malaysian adolescents. . 2014;18(7):856-861.
Agaku et al.	2015	Multiple	Article	Background: Preventing tobacco use is a key aspect of health promotion during adolescence. We assessed prevalence and impact of school-based tobacco prevention programs in 43 countries. Methods: We performed a secondary analysis of national data of students aged 13-15 years (Global Youth Tobacco Surveys) from 43 countries during 2005-2011. National surveys of the corresponding school personnel (Global School Personnel Surveys) were performed in each country during the same year as the student surveys. Data on status of enforcement of national smoke-free school policies were obtained from the 2008 and 2009 WHO MPOWER reports. Logistic regression was used to measure ecologic-level associations between school-based tobacco prevention programs and tobacco-related knowledge and behaviour among students (P < 0.05). Results: The proportion of students who were taught in class about the dangers of tobacco use during the school year ranged from 31.4% (Georgia) to 83.4% (Papua New Guinea). For every 10% increase (country level) in the proportion of teachers who reported having a tobacco prevention curriculum in their school, the odds of students reporting exposure to education in class about the dangers of tobacco increased by 6.0% (AOR = 1.06; 95% CI: 1.04-1.08). However, didactic education in class about the dangers of tobacco use was not independently associated with student current cigarette smoking behavior. Conversely, the likelihood of being a current smoker was significantly lower among students in countries with moderate/strongly enforced national smoke-free school policies compared with those in countries with poorly enforced/no national smoke-free school policies (AOR = 0.59; 95% CI: 0.45-0.76). Conclusions: Comprehensive tobacco prevention programs that include well-enforced smoke-free school policies may help reduce youth smoking.	I. T. Agaku, E. M. Obadan, O. O. Odukoya and O. Olufajo. Tobacco-free schools as a core component of youth tobacco prevention programs: a secondary analysis of data from 43 countries. European journal of public health. 2015;25(2):210-5.

Aherrera et al.	2016	Turkey	Article	Introduction In 2009, Turkey extended the smoke-free legislation to hospitality venues. Compliance, however, remains low in some hospitality venues. We identified characteristics associated with knowledge of health effects that can be prevented by the smoke-free law, the attitude towards and enforcement of the law. Methods In 2014, we conducted 400 interviews with hospitality venue owners and employees in 7 cities in Turkey. The venues were identified based on a random sampling strategy in a previous phase of the study. Results Over one-third (37.3%) of hospitality owners and employees had adequate knowledge of the health effects from secondhand smoke (SHS), 71.3% had a positive attitude towards the law and 19.5% had personally enforced the law. Participants who worked 70 hours or more per week were more likely to have a positive attitude towards the law. Older individuals, women, participants working in bars/nightclubs, venue owners receiving fines for non-compliance and current smokers were less likely to have a positive attitude towards the law. Participants working in traditional coffee houses, former smokers, and participants with a high school education or greater were more likely to enforce the law. Smokers who quit or reduced smoking because of the law were more likely to enforce the law compared with those who were not influenced by the law. Conclusions Although the attitude towards the law was positive, interventions are needed to increase knowledge on the health effects of SHS and facilitate enforcement of the law, particularly among subgroups less likely to have a positive attitude and enforce the law. © 2016 by the BMJ Publishing Group Ltd.	A. Aherrera, A. çarkoglu, M. Hayran, G. Ergör, T. Eirüder, B. Kaplan, J. Susan, L. Zheng, J. E. Cohen and A. Navas-Acien. Factors that influence attitude and enforcement of the smoke-free law in Turkey: A survey of hospitality venue owners and employees. . 2016;():.
Albuja et al.	2009	Ecuador	Article	No abstract available.	Albuja S, Daynard RA. The Framework Convention on Tobacco Control (FCTC) and the adoption of domestic tobacco control policies: the Ecuadorian experience. Tob Control. 2009;18(1):18-21. doi: 10.1136/tc.2008.026385
Alwan et al.	2009	Multiple	Report	The report provides a comprehensive overview of the evidence base for protecting people from the harms of second-hand tobacco smoke through legislation and enforcement. There is a special focus on the status of implementation of smoke-free policies, with detailed data collected for the first time ever on a global basis at both the national level and for large subnational jurisdictions. Additional analyses of smoke-free legislation were performed, allowing a more detailed understanding of progress and future challenges in this area.	WHO. WHO report on the global tobacco epidemic, 2009: Implementing smoke-free environments. Geneva: World Health Organization; 2009.
American Cancer Society	2011	Vietnam	Article	No abstract available.	Clearing the air- Communicating a smoke-free Viet Nam. American Cancer Society. 2011.
American Cancer Society	2006	Multiple	Report	No abstract available.	Enforcing Strong Smoke-free Laws: The Advocate's Guide to Enforcement Strategies. American Cancer Society / UICC Tobacco Control Strategy Planning Guide #4. 2006
American Nonsmokers' Rights Foundation	Jul-05	Multiple	Report	No abstract available.	American Nonsmokers' Rights Foundation (2012). Smokefree status of workplaces and hospitality venues around the world (July 11, 2012), 1–9. Retrieved from http://www.no-smoke.org/pdf/internationalbarsandrestaurants.pdf
Americans for Nonsmokers' Rights	2011	Multiple	Report	No abstract available.	Americans for Nonsmokers Rights. Smokefree Status of Workplaces and Hospitality Venues Around the World. Available online at: http://www.no-smoke.org/pdf/internationalbarsandrestaurants.pdf.

Au et al.	2012	China	Article	Throughout the world, cigarette smoking is a habit that causes serious health, economic, and social problems. Therefore, many countries have taken an active role to control and to ban smoking. The chronic smoking problem in China is particularly acute because China has the largest population of smokers in the world, over 300 million currently. If 30% of these smokers were to die of smoke-related diseases in the next 20 years, the impact from the more than 90 million premature deaths could be damaging to China. In addition, numerous non-smokers also experience health problems from exposure to environmental tobacco smoke. China's efforts to reduce or to ban smoking in certain public places have not been well-coordinated or enforced compared with those in other countries. Therefore, success has been minimal. Consequently, leaders in China should not be complacent about combating the serious national health problem. A multiprong approach in combination with the MPOWER policy from the World Health Organization that targets different levels of acquisition of the smoking habit must be used. Examples may include the government's reduced reliance on profits from the sale of cigarettes, the elimination of advertisements that encourage smoking among young individuals, the presentation of more graphic illustration of harmful effects from smoking on every pack of cigarettes, higher taxes/prices on cigarettes, and the implementation of enforceable bans on smoking in public places. As shown in other countries, such coordinated effort can be highly effective in the reduction of smoking and can have healthy consequenc-es.	W.W. Au, D. Su, J. Yuan. Cigarette smoking in China: public health, science, and policy. Rev Environ Health. 2012;27(1):43-9.
Au et al.	2016	China	Article	Objectives: To investigate the extent of cigarette smoking, knowledge of health hazards and attitudes towards the ban of smoking in Shantou, China, as causes for failure to control smoking. Study design: Environmental monitoring and population survey. Methods: Particulate matter (PM2.5) measure-ments were conducted in randomly selected public places (restaurants, non-alcoholic drink shops and internet bars) and exposure-related health hazards were evaluated. University students and adult citizens were randomly selected to determine their extent of cigarette smoking, knowledge of health hazards and attitude towards the ban of smoking in public places. The collected data were used to evaluate possible causes and solutions to the smoking problem. Results: From PM2.5 measurements, the average indoor to outdoor concentrations in non-smoking restaurants were 33.4 vs. 30.6 µg/m3, P > 0.05; average indoor of smoking restaurants was 350.0% higher, P < 0.05; internet bars was 395.7% higher, P < 0.05; and non-alcoholic drink shops was 650.2% higher, P > 0.001. From our survey of 1100 university students: 1) 17.5% and 7.5% were active male and female smokers, respectively; and 2) 57.5% of students would accept a smoke-ban policy. From 502 adult citizens: 1) 27.5% were active male smokers; 2) Approximately 40 and 60% had inadequate knowledge of health hazards from smoking and second-hand smoke exposure; and 3) >90% of them would accept a smoke-ban policy. Con-clusions: Our data indicate that failure to ban smoking was not caused by resistance from smokers but inadequate (national and local) government effort to educate the public and to enforce existing policy. The data suggest that development of a citizen-based approach, in collaboration with willing officials, may be highly successful in the control of cigarette smoking in China.	W. W. Au, W. Ma, Q. Zhu, H. Chen and L. Tang. Problems with cigarette smoking and attitudes towards the ban of smoking in Shantou, China. . 2016;134():46-53.
Ay et al.	2016	Turkey	Article	Objectives In Turkey, smoking has been banned in hospitality establishments since July 2009. The objective of this study was to determine noncompli-ance to the smoke-free law and its change in 2 consecutive years in enclosed spaces of hospitality venues and also to evaluate the factors associated with noncompliance. Study design This is an observational study. Methods Hospitality venues in Istanbul were visited, and data were collected through direct observation and interviews. Observation of smoking, cigarette butts or existence of ashtrays were defined as noncompliance. The survey was repeated in 2 consecutive years; the venues were visited both in 2013 and 2014. Logistic regression was used to evaluate factors associated with noncompliance. Results In 2013, 450 establishments were visited, and in the next year, 367 (81.6%) were revisited. Noncompliance for 2013 and 2014 were 49.0% and 29.7%, respectively. The highest violation was observed in bars and traditional coffeehouses. There was a significant decrease in noncompliance from 2013 to 2014 among restaurants and cafés, while such a change was not observed among bars and traditional coffeehouses. In the multivariate analysis, venues other than restaurants, venues that did not have no-smoking signs and venues which had been issued fines previously had increased probability of noncompliance. Conclusions While compliance to smoke-free law had increased significantly within 1 year, almost one third of the venues were still violating the law in 2014. The venues which were issued fines continued to violate the law. There is a need to strengthen enforcement efforts and revise the methods of enforcement and penalties in hospitality establishments.	P. Ay, E. Evrengil, M. Guner and E. Dagli. Noncompliance to smoke-free law: which hospital-ity premises are more prone?. . 2016;141():42741.
Ayo-Yusuf et al.	2014	South Africa	Article	This study sought to investigate the level of tobacco smoke pollution in eight selected popular restaurants in the capital city - Pretoria. Average PM2.5 concentrations recorded were positively correlated with active smoking density (Spearman's correlation coefficient 0.71, P=0.047), indicating that a significant source of PM2.5 in the 'nonsmoking sections' was tobacco smoke generated from designated 'smoking areas'. The median average PM2.5 con-centration (183 micro g/m3) was at least seven times higher than the WHO standard of 25 micro g/m3 set for good air quality. Furthermore, the average PM2.5 levels recorded in some restaurants were similar to levels recorded in some smoking sections in the recent Ghanaian study.	O.A. Ayo-Yusuf. Tobacco smoke pollution in the 'non-smoking' sections of selected popular restaurants in Pretoria, South Africa. Tob Control. 2014;23:193-194.
Baris et al.	2000	Multiple	Article	No abstract available.	Baris E, Brigden LW, Prindiville J, da Costa e Silva VL, Chitanondh H, Chandiwana S. Research priorities for tobacco control in developing countries: a regional approach to a global consulta-tive process. Tobacco control. 2000;9(2):217-223.

Barnoya et al.	2013	Multiple	Article	Introduction: Article 8 of the Framework Convention on Tobacco Control mandates all signatory countries to "protect citizens from exposure to tobacco smoke in workplaces, public transport and indoor public places "Even though there has been great progress in the implementation of Article 8, still most of the world population remains exposed to secondhand smoke (SHS). In this article, we sought to summarize the research that supports Article 8, where do we stand, and current research gaps and future directions. Discussion: Secondhand smoke is an established cause of heart disease and several types of cancer. Additional research is needed to reach final conclusions for diseases where evidence is only suggestive of causality. The only solution to SHS exposure in public places is banning smoking indoors. Research on the gaming industry and nightclubs, particularly in developing countries, needs to be disseminated to support their inclusion in smoke-free laws. Aside from indoor bans, additional research is needed for outdoor and multiunit housing bans and in support of measures that protect children and other vulnerable populations. The impact of smoke-free laws on other health outcomes, besides heart disease and respiratory outcomes, is another area where further research is needed. Thirdhand smoke assessment and health effects are also likely to be a topic of further research. As new tobacco products emerge, evaluating SHS exposure and effects will be vital. Conclusions: Furthering research in support of Article 8 can contribute to reach the final goal of protecting everyone from SHS exposure. (copyright) The Author 2012. Published by Oxford University Press on behalf of the Society for Research on Nicotine and Tobacco. All rights reserved.	J. Barnoya, A. Navas-Acien. Protecting the world from secondhand tobacco smoke exposure: where do we stand and where do we go from here? Nicotine Tob Res. 2013;15(4):789-804. doi: 10.1093/ntr/nts200
Barnoya et al.	2011	Guatemala	Article	Objective: In February 2009, Guatemala implemented a comprehensive smoking ban. We assessed air nicotine levels in bars and restaurants 6 months after the ban (post-ban) and compared them with levels found in 2006 (pre-ban). Methods: Exposure was estimated by passive sampling of vapor-phase nicotine using samplers (n = 50) placed for 7 working days in 10 bars and 11 restaurants in Guatemala City. Air nicotine was measured by gas chromatography, and the time-weighted average concentration in (mu)g/m3 was estimated. Employees answered a survey about smoke-free workplaces (n = 32) and compared with pre-ban (n = 37) results. Results: Nicotine was detectable in all bars pre- and post-ban. In restaurants, it was detectable in all pre- and 73% post-ban. Median nicotine concentrations in bars significantly decreased from 4.58 (mu)g/m3 (IQR, 1.71, 6.45) pre-ban to 0.28 (mu)g/m3 (IQR 0.17, 0.66) post-ban (87% decrease). In restaurants, concentrations significantly decreased from 0.58 (mu)g/m3 (IQR, 0.44, 0.71) to 0.04 (mu)g/m3 (IQR 0.01, 0.11) (95% decrease). Employees' support for a smoke-free workplace increased in the post-ban survey (from 32 to 81%, p < 0.001). Conclusion: Six months after the implementation of a smoke-free law in Guatemala, nicotine levels were significantly decreased in bars and restaurants and workers' support for the law substantially increased. (copyright) 2010 Springer Science+Business Media B.V.	J. Barnoya, M. Arvizu, M.R. Jones, J.C. Hernandez, P.N. Breysse, A. Navas-Acien. Secondhand smoke exposure in bars and restaurants in Guatemala City: before and after smoking ban evaluation. Cancer Causes & Control;2011;22(1):151-156.
Barnoya et al.	2003	Latin/ South America	Article	For more than 20 years the tobacco industry has considered secondhand smoke to be a threat to its viability. In this article, we describe why secondhand smoke is important to tobacco control and how the tobacco industry's "Latin Project" sought to prevent the creation of smoke-free workplaces and public places in Central and South America. Eliminating secondhand smoke exposure not only reduces the risk of cardiovascular and other diseases, but also creates an environment that substantially reduces smoking and cigarette consumption among smokers. The "Latin Project" was initiated in 1991 by Philip Morris and British American Tobacco and managed by the law firm Covington & Burling. The project assembled a network of well-placed physicians and scientists to divert the attention away from secondhand smoke toward other indoor air pollutants. As proven in Central and South America, the tobacco industry has manipulated the secondhand smoke issue in order to avoid the development of smoke-free environments. Sub-Saharan Africa, facing an epidemiologic transition similar to the one experienced by Central and South America, should be aware of tobacco industry tactics. Further delay in implementing smoke-free environments will only increase the burden of cardiovascular disease in both areas of the world.	J. Barnoya, S.A. Glantz. The tobacco industry and secondhand smoke: Lessons from Central and South America. Ethnicity and Disease. 2003;13(2 SUPPL. 2):S2-88-S2-90.
Barnoya et al.	2016	Guatemala	Article	Background: Smoke-free environments decrease smoking prevalence and consequently the incidence of heart disease and lung cancer. Due to issues related to poor enforcement, scant data is currently available from low/middle income countries on the long-term compliance to smoke-free laws. In 2006, high levels of secondhand smoke (SHS) were found in bars and restaurants in Guatemala City. Six months after a smoking ban was implemented in 2009, levels significantly decreased. However, in 2010, poor law compliance was observed. Therefore, we sought to assess long-term compliance to the ban using SHS measurements. Methods: In 2014 we assessed SHS exposure using airborne nicotine monitors in bars (n = 9) and restaurants (n = 12) for 7 days using the same protocol as in 2006 and in 2009. Nicotine was measured using gas-chromatography (mug/m(3)) and compared to levels pre- (2006) and post-ban (2009). Employees responded to a survey about SHS exposure, perceived economic impact of the ban and customers' electronic cigarette use. In addition, we estimated the fines that could have been collected for each law infringement. Results: Most (71 %) venues still have a smoking section, violating the law. The percentage of samples with detectable nicotine concentrations was 100, 85 and 43 % in 2006, 2009 and 2014, respectively. In bars, median (25(th) and 75(th) percentiles) nicotine concentrations were 4.58 mug/m(3) (1.71, 6.45) in 2006, 0.28 (0.17, 0.66) in 2009, and 0.59 (0.01, 1.45) in 2014. In restaurants, the corresponding medians were 0.58 mug/m(3) (0.44, 0.71), 0.04 (0.01, 0.11), and 0.01 (0.01, 0.09). Support for the law continues to be high (88 %) among bar and restaurant employees. Most employees report no economic impact of the law and that a high proportion of customers (78 %) use e-cigarettes. A total of US$50,012 could have been collected in fines. Conclusions: Long-term compliance to the smoking ban in Guatemala is decreasing. Additional research that evaluates the determinants of non-compliance is needed and could also contribute to improve enforcement and implementation of the smoke-free law in Guatemala.	J. Barnoya, J. C. Monzon, P. Briz and A. Navas-Acien. Compliance to the smoke-free law in Guatemala 5-years after implementation. BMC public health. 2016;16():318.
Barnoya et al.	2006	Multiple	Article	No abstract available.	Barnoya J, Glantz SA. The tobacco industry's worldwide ETS consultants project: European and Asian components. European journal of public health. 2006;16(1):69-77. doi: 10.1093/eurpub/cki044

Barnoya et al.	2002	Multiple	Article	No abstract available.	Barnoya J, Glantz S. Tobacco industry success in preventing regulation of secondhand smoke in Latin America: the "Latin Project". Tobacco control. 2002;11(4):305-314.
Baron-Epel et al.	2012	Multiple	Article	Background: The latest amendment to the ban on smoking in public places in Israel was implemented in 2007, adding pubs and bars (P&B) to the list of public places in which smoking is prohibited. However, smoking in most P&B continued. The aim of the study was to identify the theoretically plausible reasons for the partial success of a public ban on smoking in P&B settings. Explanations provided by P&B owners were interpreted as probable causal factors based on the Behavioral Ecological Model (BEM). Methods: Qualitative interviews were performed with 36 P&B owners in Tel-Aviv and 18 Israeli towns and cities of various population size. Results: P&B owners reported a variety of situational factors (i.e., contingencies) and reinforcers as likely explanations of the partial failure of the legislated ban on smoking in public places, particularly P&B. The major reinforcers for non-adherence with the law were no or low frequency of inspections and low penalties from authorities. P&B owners also feared loss of customers and revenue if bans were enforced in their own establishment but not in competing establishments. Finally, owners reported social norms prevailing among some Israeli patrons supporting smoking in P&B settings, in part to express opposition to the new law. Conclusions: Qualitative assessment can uncover probable social situations that operate to prevent greater adherence to smoking bans. The results warrant confirmation by quantitative analyses. Policies with mandated inspections and penalty requirements that are implemented in all bars without prejudice could lead to greater adherence to smoking bans. Positive reinforcing consequences that encourage adherence (such as publicity and support from non-smokers) would be more likely to generate both greater adherence to the policy and good will toward the government. Principles of behavior outlined in the BEM offer guidance for designing quantitative confirmation analyses of future bans.	Baron-Epel O, Satran C, Cohen V, Drach-Zehavi A, Hovell MF. Challenges for the smoking ban in Israeli pubs and bars: analysis guided by the behavioral ecological model. Israel J Health Policy Res. 2012;1(1):28. doi: 10.1186/2045-4015-1-28
Bhat et al.	2015	India	Article	Background: The aim of this study was to evaluate the effect of anti-smoking legislation in public places and public support for smoking ban. Methods: A cross-sectional survey was conducted in public places such as market, bus/railway station, workplaces and hospitals in Udaipur, India. Informed consent was obtained. The questionnaire comprised of details about their agreement with the current anti-smoking legislation and their views on the support for smoking ban in public places. FINDINGS: The study was conducted among 314 individuals of whom 255 (81.2%) supported the general ban on smoking in public places. Non-smokers (54.4%) agreed more than smokers that the introduction of legislation would create healthier environment ($P < 0.001$), second hand smoke is serious threat to health ($P < 0.001$), more implementation needed for current antismoking legislation ($P < 0.001$). Smokers (45.5%) agreed more than non-smokers that everyone has the right to smoke in public places ($P < 0.001$). Conclusion: Local support for a ban on smoking in public places in Udaipur is high. But there is a lack of enforcing mechanism in support of the legislation and hence complete smoking ban along with strong enforcement may provide better results.	N. Bhat, S. Oza, J. J. Reddy, R. Mitra, P. Rahul and S. Singh. Effect of Anti-Smoking Legislation in Public Places. Addiction & health. 2015;7(42737):87-91.
Bilir et al.	2013	Turkey	Article	In 2003 World Health Assembly adopted an international treaty on tobacco control; FCTC, Framework Convention on Tobacco Control. Five year later World Health Organization (WHO) declared the six effective approach for tobacco control, under the name of MPOWER. In the following years, WHO evaluated the level of implementation of MPOWER criteria in the countries. In this article, how Turkey implemented these six criteria will be discussed. Monitor tobacco use and prevention policies: Monitoring of tobacco use prevalence has been successfully monitored in Turkey through Global Adult Tobacco Survey, Global Youth Tobacco Survey and Health Professionals Tobacco Use Survey. Nevertheless, monitoring of tobacco industry activities was not successfully implemented. Protect people from tobacco smoke: Smoking was banned in most of the indoor public places in Turkey since 1996, and Turkey became a complete smoke-free country by the exposion of smoke-free places including the hospitality workplaces in 2008. Offer help to quit tobacco use: Although smoking cessation services has been a bit late in Turkey, availability of smoking cessation drugs and the establishment of free quitline services made Turkey successful in this regard. Warn about the dangers of tobacco: Since 1996, all TV channels have a duty of broadcasting programs on harms of tobacco use, not less than 90 minutes in a month and it has been implemented successfully. Additionally written messages indicating harms of tobacco has been printed on the packs since 1988 and pictures was added in 2010. But since the average surface area covered by the messages in less than 50% of the total surface of the pack, Turkey was not regarded as to meet the requirement. Enforce bans on tobacco advertising, promotion and sponsorship: All kinds of tobacco advertisement and promotion was banned by the Law in 1996. But the tobacco products was not in closed boxes at the sales points. Turkey was not found as successful in this regard. Raise taxes on tobacco: Total tax on the tobacco products is more than 75% level which was recommended by WHO, Turkey meets this criteria. In conclusion, Turkey was regarded as meeting all the MPOWER criteria except the warning about the dangers of tobacco and enforcement of the advertisement ban. In conclusion, Turkey was regarded as meeting all the MPOWER criteria except the warning about the dangers of tobacco and enforcement of the advertisement ban.	N. Bilir, H. Ozcebe. How Turkey meets MPOWER criteria? TAF Preventive Medicine Bulletin. 2013;12(1):41649.

Bilir et al.	2012	Turkey	Article	Objective: In this descriptive study, it was aimed to assess attitudes and behaviours of some taxi-drivers in Ankara on the smoking ban in cabs under the Law of Preventing the Harmful effects of Tobacco Products. Material and Methods: Face to face interviews were done with 277 taxi-drivers by visiting taxi stations in Alti{dotless}ndag and Cankaya districts. Additionally, breath carbon monoxide levels were measured in some drivers after obtaining their informed consent. As a part of this study, the actual smoking status of the drivers and/or clients were recorded by observing the travelling taxies on the junctions of the main streets of the two districts of Ankara. Results: Sixty-four percent of taxi drivers (177 of 277) were current smokers. The majority of these current smokers (108 of 177; 61.0%) reported that they also smoked in the cabs. While more than half of the respondents (55.2%) supported the smoking ban in the cabs, one third (33.2%) of the drivers strongly disagreed with this legislation. Among the taxi drivers, there was an agreement on supporting the smoking bans in certain public places like court halls and the town bus stations (80.2% and 73.6% respectively), but on the other hand, they s were less supportive of the bans in cafes and restaurants, particularly serving alcoholic drink (41.5% and 32.9% respectively). Non-smoker drivers supported the smoking ban legislations more. Conclusion: Findings showed that compliance with the items of the Law which ban smoking in the cabs, were not sufficient. In daily life many traffic policemen are responsible for the regulation of the traffic. It will be efficacious in the eradication of smoke from the cabs, if these traffic police are informed about their authorization and duty on the tobacco control, including fining the taxi drivers who ignore the smoking ban legislation.	N. Bilir, M.S. Yardim, M. Alisik, O. Arpat, Y. Atalay, B. Aydogan. Assessment of attitudes and behaviours of taxi-drivers in Ankara on the smoking ban in cabs. Turk Toraks Dergisi. 2012;13(4):141-145.
Bump et al.	2013	Multiple	Article	Tobacco is already the world's leading cause of preventable death, claiming over 5 million lives annually, and this toll is rising. Even though effective tobacco control policies are well researched and widely disseminated, they remain largely unimplemented in most low- and middle-income countries (LMICs). For the most part, control attempts by advocates and government regulators have been frustrated by transnational tobacco companies (TTCs) and their supporters. One reason tobacco is so difficult to control is that its political economy has yet to be adequately understood and addressed. We conducted a review of the literature on tobacco control in LMICs using the databases PubMed, EconLit, PsychInfo and AGRICOLA. Among the over 2500 papers and reports we identified, very few explicitly applied political economy analysis to tobacco control in an LMIC setting. The vast majority of papers characterized important aspects of the tobacco epidemic, including who smokes, the effects of smoking on health, the effectiveness of advertising bans, and the activities of TTCs and their allies. But the political and economic dynamics of policy adoption and implementation were not discussed in any but a handful of papers. To help control advocates better understand and manage the process of policy implementation, we identify how political economy analysis would differ from the traditional public health approaches that dominate the literature. We focus on five important problem areas: information problems and the risks of smoking; the roles of domestic producers; multinational corporations and trade disputes in consumption; smuggling; the barriers to raising taxes and establishing spatial restrictions on smoking; and incentive conflicts between government branches. We conclude by discussing the political economy of tobacco and its implications for control strategies.	J. B. Bump, M. R. Reich. Political economy analysis for tobacco control in low- and middle-income countries. Health Policy Plan. 2013;28(2):123-33.
Burns	1992	Multiple	Article	No abstract available.	Burns DM, Axelrad R, Bal D, et al. Report of the Tobacco Policy Research Study Group on smoke-free indoor air policies. Tobacco control. 1992;1:S14-S18.

Callinan et al.	2010	Multiple	Article	Background: Smoking bans have been implemented in a variety of settings, as well as being part of policy in many jurisdictions to protect the public and employees from the harmful effects of secondhand smoke (SHS). They also offer the potential to influence social norms and smoking behaviour of those populations they affect. Objectives: To assess the extent to which legislation-based smoking bans or restrictions reduce exposure to SHS, help people who smoke to reduce tobacco consumption or lower smoking prevalence and affect the health of those in areas which have a ban or restriction in place. SEARCH STRATEGY: We searched the Cochrane Tobacco Addiction Group Specialised Register, MEDLINE, EMBASE, PsycINFO, CINAHL, Conference Paper Index, and reference lists and bibliographies of included studies. We also checked websites of various organisations. Date of most recent search; July 1st 2009. SELECTION CRITERIA: We considered studies that reported legislative smoking bans and restrictions affecting populations. The minimum standard was having a ban explicitly in the study and a minimum of six months follow-up for measures of smoking behaviour. We included randomized controlled trials, quasi-experimental studies (i.e. non-randomized controlled studies), controlled before and after studies, interrupted-time series as defined by the Cochrane Effective Practice and Organization of Care Group, and uncontrolled pre- and post-ban data. DATA COLLECTION AND ANALYSIS: Characteristics and content of the interventions, participants, outcomes and methods of the included studies were extracted by one author and checked by a second. Because of heterogeneity in the design and content of the studies, we did not attempt a meta-analysis. We evaluated the studies using qualitative narrative synthesis. MAIN Results: There were 50 studies included in this review. Thirty-one studies reported exposure to secondhand smoke (SHS) with 19 studies measuring it using biomarkers. There was consistent evidence that smoking bans reduced exposure to SHS in workplaces, restaurants, pubs and in public places. There was a greater reduction in exposure to SHS in hospitality workers compared to the general population. We failed to detect any difference in self-reported exposure to SHS in cars. There was no change in either the prevalence or duration of reported exposure to SHS in the home as a result of implementing legislative bans. Twenty-three studies reported measures of active smoking, often as a co-variable rather than an end-point in itself, with no consistent evidence of a reduction in smoking prevalence attributable to the ban. Total tobacco consumption was reduced in studies where prevalence declined. Twenty-five studies reported health indicators as an outcome. Self-reported respiratory and sensory symptoms were measured in 12 studies, with lung function measured in five of them. There was consistent evidence of a reduction in hospital admissions for cardiac events as well as an improvement in some health indicators after the ban. AUTHORS' Conclusions: Introduction of a legislative smoking ban does lead to a reduction in exposure to passive smoking. Hospitality workers experienced a greater reduction in exposure to SHS after implementing the ban compared to the general population. There is limited evidence about the impact on active smoking but the trend is downwards. There is some evidence of an improvement in health outcomes. The strongest evidence is the reduction seen in admissions for acute coronary syndrome. There is an increase in support for and compliance with smoking bans after the legislation.	J. E. Callinan;A. Clarke;K. Doherty;C. Kelleher. Legislative smoking bans for reducing secondhand smoke exposure, smoking prevalence and tobacco consumption. Cochrane Database Syst Rev. 2010;(4):CD005992.
Campelo et al.	2011	Brazil	Report	No abstract available.	Campelo DG. WHO smoke-free city case study: Recife breathing better: case study of smoke-free policy. Geneva: World Health Organization;2011.
Casaubón et al.	2011	Mexico	Report	No abstract available.	Casaubón ME, Ahued-Ortega A, Robles-García R, et al. WHO smoke-free city case study: Towards 100% smoke-free environment: the case study of Mexico City, Mexico. Geneva: World Health Organization;2011.
Casaubón et al.	2011	India	Report	No abstract available.	Arul R, Angelis S, Ansari SAT, Kavitha G. WHO smoke-free city case study: Towards breathing clean air in Chennai - A smoke-free city case study. Geneva: World Health Organization;2011.

Chang et al.	2009	Mongolia	Article	Objectives: Exposure to second-hand smoke (SHS) is widespread in restaurants in Ulaanbaatar, the capital city of Mongolia. While a smoke-free policy is the most effective way of protecting restaurant workers and customers from SHS, this has not been well accepted in Mongolia. Furthermore, little is known about restaurants' attitude toward the smoke-free policy. Methods: A cross-sectional survey directed to restaurant owners or managers was conducted in 475 representative restaurants in Ulaanbaatar. Face-to-face interviews using a questionnaire and on-site observation were performed. Results: Only 29.3% of the restaurants claimed to prohibit smoking; none of the remaining had any protection toward SHS, and half of the restaurants estimated that more than 20% of customers would smoke inside. None of them had visible "no smoking" signs and the majority never received complaints about SHS. Despite the generally high level of knowledge of the health effects of SHS, of the 336 restaurants that were not smoke free, only 25.9% expressed that they planned to take action in the near future. By contrast, 87.8% of restaurants would support the government if it asked all restaurants to ban smoking. Multivariate analysis identified that restaurants having menus in foreign languages, selling cigarettes and predicting business decline were less likely to support the government smoke-free policy. Conclusions: This survey demonstrates that restaurants owners and managers were reluctant to take action on their own, but would support government policy. The government can assume a stronger role first by revising the law on tobacco control following the Framework Convention on Tobacco Control guideline.	S. H. Chang;V. Delgermaa;K. Mungun-Ulzii;N. Erdenekhuu;E. Odkhuu;S. L. Huang. Support for smoke-free policy among restaurant owners and managers in Ulaanbaatar, Mongolia. Tob Control. 2009;18(6):479-484.
Crosbie et al.	2011	Mexico	Article	Objective: To describe the approval process and implementation of the 100% smokefree law in Mexico City and a competing federal law between 2007 and 2010. Methods: Reviewed smokefree legislation, published newspaper articles and interviewed key informants. Results: Strong efforts by tobacco control advocacy groups and key policymakers in Mexico City in 2008 prompted the approval of a 100% smokefree law following the WHO FCTC. As elsewhere, the tobacco industry utilised the hospitality sector to block smokefree legislation, challenged the City law before the Supreme Court and promoted the passage of a federal law that required designated smoking areas. These tactics disrupted implementation of the City law by causing confusion over which law applied in Mexico City. Despite interference, the City law increased public support for 100% smokefree policies and decreased the social acceptability of smoking. In September 2009, the Supreme Court ruled in favour of the City law, giving it the authority to go beyond the federal law to protect the fundamental right of health for all citizens. Conclusions: Early education and enforcement efforts by tobacco control advocates promoted the City law in 2008 but advocates should still anticipate continuing opposition from the tobacco industry, which will require continued pressure on the government. Advocates should utilise the Supreme Court's ruling to promote 100% smokefree policies outside Mexico City. Strong advocacy for the City law could be used as a model of success throughout Mexico and other Latin American countries.	E. Crosbie;E. M. Sebrie;S. A. Glantz. Strong advocacy led to successful implementation of smokefree Mexico City. Tob Control. 2011;20(1):64-72.
Crosbie et al.	2016	Costa Rica	Article	Objective To analyse the process of implementing and enforcing smoke-free environments, tobacco advertising, tobacco taxes and health warning labels from Costa Rica's 2012 tobacco control law. Method Review of tobacco control legislation, newspaper articles and interviewing key informants. Results Despite overcoming decades of tobacco industry dominance to win enactment of a strong tobacco control law in March 2012 consistent with WHO's Framework Convention on Tobacco Control, the tobacco industry and their allies lobbied executive branch authorities for exemptions in smoke-free environments to create public confusion, and continued to report in the media that increasing cigarette taxes led to a rise in illicit trade. In response, tobacco control advocates, with technical support from international health groups, helped strengthen tobacco advertising regulations by prohibiting advertising at the point-of-sale (POS) and banning corporate social responsibility campaigns. The Health Ministry used increased tobacco taxes earmarked for tobacco control to help effectively promote and enforce the law, resulting in high compliance for smokefree environments, advertising restrictions and health warning label (HWL) regulations. Despite this success, government trade concerns allowed, as of December 2015, POS tobacco advertising, and delayed the release of HWL regulations for 15 months. Conclusions The implementation phase continues to be a site of intensive tobacco industry political activity in low and middle-income countries. International support and earmarked tobacco taxes provide important technical and financial assistance to implement tobacco control policies, but more legal expertise is needed to overcome government trade concerns and avoid unnecessary delays in implementation. © 2016 by the BMJ Publishing Group Ltd.	E. Crosbie, P. Sosa and S. A. Glantz. The importance of continued engagement during the implementation phase of tobacco control policies in a middle-income country: The case of Costa Rica. . 2016;():.
Cummings	2009	Multiple	Report	No abstract available.	K. M. Cummings, C. T. Orleans. Policies to achieve a smoke-free society- A research agenda for 2010-2015. Robert Wood Johnson Foundation. 2009.
Dawson et al.	2009	India	Article	No abstract available.	Dawson J, Singh RJ. Smokefree City: Chandigarh. Edinburgh: International Union Against Tuberculosis and Lung Disease;2009.
Dawson et al.	2009	Mexico	Article	No abstract available.	Dawson J, Romo J, Molinari M, Garrido L. Smokefree city: Mexico DF. Extended Report. Edinburgh: International Union Against Tuberculosis and Lung Disease;2009.

Dawson et al.	2009	Mexico	Article	No abstract available.	Dawson J, Romo J, Molinari M, Garrido L. Smokefree city: Mexico DF. Edinburgh: International Union Against Tuberculosis and Lung Disease;2009.
Donaldson et al.	2011	India	Article	No abstract available.	Donaldson EA, Waters HR, Arora M, Varghese B, Dave P, Modi B. A cost-effectiveness analysis of India's 2008 prohibition of smoking in public places in Gujarat. Int J Environ Res Public Health. 2011;8(5):1271–1286. doi:10.3390/ijerph8051271
Drope et al.	2010	African countries	Article	The public health rewards of smoke-free policies are well documented. But in their enthusiasm to achieve such policies, public health advocates and policymakers frequently underestimate the political complexity of passing laws, and then implementing and enforcing them. Using 12 African countries as the focus of discussion, this research examines the basic political process for and the barriers to achieving smoke-free policies. Moreover, in addition to the obstacles, it examines why some countries have been experiencing comparatively more success in the smoke-free policy area. The findings of the research suggest strongly that the presence of a vigorous tobacco control civil society movement, some will on the part of government institutions, and active research support contribute significantly to successful smoke-free policies. It is also apparent that the emerging battle fronts in smoke-free policies are in the areas of implementation and enforcement, and while similar variables that affect the passing of new laws also condition these outcomes, there are the added distinct challenges of policy fatigue and additional resource constraints. (copyright) 2010 Published by Elsevier Ltd. on behalf of World Heart Federation.	J. M. Drope. The politics of smoke-free policies in developing countries: Lessons from Africa. CVD Prevention and Control. 2010;5(3):65-73.
Efroymson et al.	2009	Multiple	Report	No abstract available.	Efroymson D, Alam SM. Enforcement of tobacco control law: A guide to the basics. Ottawa, ON: HealthBridge;February 2009.
Efroymson et al.	2006	Multiple	Report	No abstract available.	Efroymson D, Alam SM. Using media and research for advocacy: Low cost ways to increase success. Ottawa, ON: HealthBridge;February 2009.
Erika et al.	2011	Albania	Article	Background: The implementation of smoking bans in Albania has been quite difficult, especially because of the high smoking rates. The aim of this study was to utilise the general theory of deterrence (GTD) to understand non-compliance with smoke-free policies in public places. Participants were 333 university student smokers (Mage ¼ 21.2 years). Measures: A self-report questionnaire was administered to assess variables from the GTD, attitudes and norms. Results: Smokers reported high noncompliance rates, strong social norms but weak attitude towards non-compliance with smoke-free policies. Furthermore, non-compliance was predicted by weak legal, moral and social deterrents. Discussion: The findings suggest that although Albanian smokers were negative towards noncompliance, they still violate smokefree policies in public settings, mainly as a result of weak legal and social deterrents. From a practical perspective, strengthening the barriers to noncompliance is particularly important (e.g. campaigns, frequent controls, fines, etc.).	M. Erika;E. J. Richard;R. Angelos;L. Lambros. Understanding non-compliance with smoke-free polices in public places in Albania: A case of weak social deterrents?. Psychology & Health. 2011;26():174-174.
Fong et al.	2015	China	Article	Background: China is the world's largest consumer of tobacco, with hundreds of millions of people exposed daily to secondhand smoke (SHS). Comprehensive smoke-free policies are the only effective way to protect the population from the harms of SHS. China does not have a comprehensive national smoke-free law but some local-level regulations have been implemented. Objective: To evaluate local level smoke-free regulations across 7 cities in China by measuring the prevalence of smoking in public places (workplaces, restaurants and bars), and support for smoke-free policies over time. Methods: Data were from Waves 2 to 4 of the International Tobacco Control (ITC) China Survey (2007-2012), a face-to-face cohort survey of approximately 800 smokers in each of 7 cities in mainland China. Multivariate logistic regression models estimated with generalised estimating equations were used to test the changes in variables over time. Results: As of 2012, over three-quarters of respondents were exposed to smoking in bars; more than two-thirds were exposed to smoking in restaurants and more than half were exposed to smoking in indoor workplaces. Small decreases in the prevalence of smoking were found overall from Waves 2 to 4 for indoor workplaces, restaurants and bars, although the decline was minimal for bars. Support for complete smoking bans increased over time for each venue, although it was lowest for bars. Conclusions: Existing partial smoking bans across China have had minimal impact on reducing smoking in public places. A strongly enforced, comprehensive national smoke-free law is urgently needed in order to achieve greater public health gains.	G. T. Fong, G. Sansone, M. Yan, L. Craig, A. C. Quah and Y. Jiang. Evaluation of smoke-free policies in seven cities in China, 2007-2012. Tobacco control. 2015;24 Suppl 4():iv14-20.

Fong et al.	2006	Multiple	Article	No abstract available.	Fong GT, Cummings KM, Shopland DR; ITC Collaboration. Building the evidence base for effective tobacco control policies: the International Tobacco Control Policy Evaluation Project (the ITC Project). Tob Control. 2006;15 Suppl 3(Suppl 3):iii1–iii2. doi:10.1136/tc.2006.017244
Fong et al.	2006	Multiple	Article	No abstract available.	Fong GT, Cummings KM, Borland R, et al. The conceptual framework of the International Tobacco Control (ITC) Policy Evaluation Project. Tobacco control. 2006;15 Suppl 3:iii3-11. doi: 10.1136/tc.2005.015438
Gaswami et al.	2011	India	Report	No abstract available.	Goswami H. WHO smoke-free city case study: Experience of Chandigarh as a smoke-free city. Geneva: World Health Organization;2011.
Global Smokefree Partnership	2008	Multiple	Report	No abstract available.	Global Smokefree Partnership. Global voices: Working for smokefree air, 2008 Status report. Coordinated by the American Cancer Society and the Framework Convention Alliance. 2008.
Global Smokefree Partnership	2009	Multiple	Report	No abstract available.	Global Smokefree Partnership. Global voices status report 2009: Rebutting the tobacco industry, winning smokefree air. Coordinated by the American Cancer Society and the Framework Convention Alliance;2009.
Global Smokefree Partnership	2009	Multiple	Report	No abstract available.	Global Smokefree Partnership. Smokefree air law enforcement: Lessons from the field. Coordinated by the American Cancer Society and the Framework Convention Alliance;2009.
Global Smokefree Partnership	2010	Multiple	Report	No abstract available.	Global Smokefree Partnership. Status Report on Article 8. 2010.

Goel et al.	2013	India	Article	Background: Compliance survey of smoke-free law is an effective means of measuring progress towards a smoke-free society. They also help policy makers to take action where strengthening measures are required. India has a comprehensive tobacco control law known as Cigarettes and Other Tobacco Products Act (COTPA 2003) which prohibits smoking in public places and requires display of 'No smoking' signages with proper specifications at conspicuous points. However, its implementation and enforcement are still a matter of concern. AIMS AND Objectives: To ascertain the level of compliance with smoke-free law in public places of a district of North India. METHODOLOGY: A cross sectional study was conducted in the months of November-December 2011 in district SAS Nagar Mohali of North India. The public places including hotels/restaurants/bars/shopping malls, government offices, educational institutions, healthcare facilities and transit stations were surveyed. The study tool was adapted from the guide on 'Assessing compliance with smoke-free law' developed jointly by the Campaign for Tobacco Free Kids, Johns Hopkins Bloomberg School of Public Health and International Union against Tuberculosis and Lung Disease. Results: The overall compliance rate towards section 4 of COTPA was 92.3%. No active smoking was observed in 94.2% of the public places. In 90% of the public places 'No Smoking' signage were displayed as per COTPA. Health and educational institutions had maximum compliance with the smoke-free law while transit sites showed the least compliance. Conclusions: Compliance to the smoke-free law was high in the study.	S. Goel;K. Ravindra;R. J. Singh;D. Sharma. Effective smoke-free policies in achieving a high level of compliance with smoke-free law: experiences from a district of North India. Tob Control. 2013;():.
Goel et al.	2014	India	Article	Setting: Compliance assessment surveys are cost-effective means of assessing smokefree status in a jurisdiction. Assigning weights to assessment criteria (indicators) can also inform law implementers and policy makers about the effectiveness of the enforcement of smokefree rules. Objective: To develop a standardised measure for compliance surveys using the Delphi method in India. Design: Tobacco control experts from India comprising different constituencies and jurisdictions met for a halfday workshop in August 2012 to deliberate on how weights can be assigned to criteria for smokefree status. Using the Delphi method, the relevance and ranking of criteria from an existing protocol for measuring compliance was evaluated. Results: Consensus was reached on all five compliance survey indicators through three rigorous rounds of discussion. The highest priority was assigned to the absence of the act of smoking in public places (33%), followed by the display of no-smoking signage in public places (32%), absence of cigarette butts or bidi stubs (15%), absence of smoking aids (10%) and absence of tobacco smoke and ash (10%). Conclusion: Tobacco control advocates can effectively inform local policy makers using weights that prioritise directed enforcement and targeted interventions, which in turn will ensure stronger compliance and sustainable smokefree settings. (copyright) 2014 The Union.	S. Goel;R. Kumar;P. Lal;D. Sharma;R. J. Singh. Refining compliance surveys to measure the smokefree status of jurisdictions using the Delphi method. Public Health Action. 2014;3(4):342-345.
Goel et al.	2014	India	Article	Context: A growing number of cities, districts, counties and states across the globe are going smoke-free. While an Indian national law namely Cigarettes and Other Tobacco Products Act (COTPA) exists since 2003 and aims at protecting all the people in our country; people still smoke in public places. Aim: This study assessed knowledge and perceptions about smoking, SHS and their support for Smoke-free laws among people residing in Mohali district, Punjab. Materials and Methods: This cross-sectional study was conducted in Mohali district of Punjab, India. A sample size of 1600 people was obtained. Probability Proportional to Size technique was used for selecting the number of individuals to be interviewed from each block and also from urban and rural population. Statistical Analysis Used: We estimated proportions and tested for significant differences by residence, smoking status, literacy level and employment level by means of the chi-square statistics. Statistical software SPSS for Windows version 20 was used for analysing data . Results: The overall prevalence of current smoking among study participants was 25%. Around 96% were aware of the fact that smoking is harmful to health, 45% viewed second-hand smoke to be equally harmful as active smoking, 84.2% knew that smoking is prohibited in public places and 88.3% wanted the government to take strict actions to control the menace of public smoking. Multivariate logistic regression analysis showed that people aged 20 years and above, unemployed, urban, literate and non-smokers had significantly better perception towards harms of smoking. The knowledge about smoke free provisions of COTPA was significantly better among males, employed individuals, urban residents, and literate people. Conclusions: There was high knowledge about deleterious multi-dimensional effects of smoking among residents and a high support for implementation of COTPA. Efforts should be taken to make Mohali a "smoke-free district".	S. Goel, R. J. Singh, S. D and S. A. Public opinion about smoking and smoke free legislation in a district of North India. Indian journal of cancer. 2014;51(3):330-334.
Gruer et al.	2012	Multiple	Article	Article 8 of the World Health Organization Framework Convention on Tobacco Control (2005) requires all signatory countries to adopt measures to protect people from tobacco smoke in indoor workplaces, indoor public places, public transport and other public places as appropriate. The aims of this symposium were to review progress across the world, to assess the evidence for the impact of legislation on health, and to identify the continuing challenges in making universal protection a reality. There was agreement that even in countries where strict legislation is enforced, many children continue to be dangerously exposed to parental second-hand smoke in the womb, the home and private cars. The importance of using accurate estimates of the burden of disease caused by second-hand smoke was agreed, in order to present an unassailable case for legislation and enforcement. (copyright) 2012 The Royal Society for Public Health.	L. Gruer;E. Tursan d'Espaignet;S. Haw;E. Fernandez;J. Mackay. Smoke-free legislation: Global reach, impact and remaining challenges. Public Health. 2012;126(3):227-229.

Gvinianidze et al.	2012	Georgia	Article	Before year 2008 smoking was partially restricted in cafes and restaurants of Georgia. In 2009 Georgian Parliament adopted amendment in law "Concerning Tobacco Control" and strengthened partial restriction in cafes and restaurants, namely required that 50% of territory of those facilities must be smoke free. To observe status of implementation of tobacco control legislation in field of prohibition/restriction of smoking in cafes and restaurants conducted observation of those facilities and in-depth interviews of their owners/staff. Observation in cafes and restaurants were done in big regional centers of Georgia, namely in Tbilisi (Capital), Telavi, Kutaisi, Batumi, Rustavi, Gori, Akhaltsikhe and Zugdidi. At all 176 cafes/restaurants were observed (22,4% of all registered cafes/restaurants in Georgia). For qualitative part of the study 1-2 persons from staff of the cafe or restaurant or its owner available during the observation was interviewed. Field work was done during 2011-2012. It must be mentioned that during this period was not observed any important change in legislation and/or enforcement of smoking ban/restrictions in those facilities. Study instrument were guide for observer that contained two parts - observational and open-ended questions for owners and staff. Observation of cafes/restaurants in Georgia shows that 89,8% of them violate existing restriction on smoking. All restaurants and 85% of cafes violate the law. Only 18 (10,2%) cafes are in compliance with the legislation and all of them have total ban. Despite to the fact that more than 50% of observed cafes/restaurants were located in Tbilisi, absolute majority (88,1%) of smoke free facilities are in regions (mainly in Kutaisi, Zugdidi and Gori). Qualitative study of owners/staff of the facilities found factors that probably are influential in determination of smoking status of cafes/restaurants. Namely, decisions on those kinds of issues are made by owners according to business interest and private attitude. Because of absence of enforcement activities requirement of tobacco control legislation is not taken into consideration. Existing partial restriction doesn't have support from great majority of cafes/restaurants, because it is difficult to implement. At the same time part of owners/staff don't support any type of ban or restriction, because of their fear to loose smoker clients. At that qualitative study and observation shows that this opinion is assumption and not on practice or evidence based argument. Despite to this assumption, total ban on smoking in cafes and restaurants is understood as "coming legislation" and main requirement to this possible development is to provide "ban for all" and information of general public.	K. Gvinianidze;G. Bakhturidze;G. Magradze. Study of implementation level of tobacco restriction policy in cafes and restaurants of Georgia. Georgian Med News. 2012;(206):57-63.
Heydari et al.	2009	Iran	Article	Background: Creating smoke-free public places is essential to protect non-smokers and it also motivates smokers to quit. Considering the smoking ban law in public places of Iran and weakly enforcement of this law, we decided to study the circumstances in Tehran. Methods: It was a cross-sectional study which has been done during 2009. Firstly, Tehran's geographic map was divided into 3 areas of north, center and south and secondly 10 spots in each area were selected randomly. By reaching to that addresses easy sampling was done and going straightly toward the right side to collect enough cases. The indicators including "the existence of smoking ban signs, ashtrays and smoking areas and also the way of showing objection against smoking" were questioned and observed. Results: 458 restaurants and food courts were studied. In 60 places (13%) there were no smoking ban signs. In 140 places (30%) there were ash trays and in 54 places (12%) there were smoking areas. In 132 places (29%) the in-charge people had no objection against smoking in restaurants. All the cases were aware of smoking ban law in public places. More than 50% considered it as a useful law and 106 restaurants (23%) believed that the law enforcement would increase the number of customers. There was no statistically relationship between the place and working year of restaurants and law enforcement but it was shown a statistically relationship between smoking ban signs and showing objection against smoking and increasing the number of customers (p=0.000). Conclusion: In spite of the fact that it has been a long time since the law adaptation, but it has not been fully implemented, so it is necessary to reinforce the law.	G. Heydari;A. Ramezankhani;F. Talischi;M. R. Masjedi. Is smoke free legislation enforcing completely in Tehran 2009?. J of Medical Council of Islamic Republic of Iran. 2009;27:Pe423-En509.
Heydari et al.	2013	Multple	Article	The aim of this cross-sectional study was to quantify the implementation of MPOWER tobacco control policies among Eastern Mediterranean Region countries. Information was obtained from the 2011 WHO MPOWER report. A checklist was designed and its scoring was agreed by Iranian and international tobacco control specialists. Seven questions were scored from 0–4 and 3 from 0–3. The 22 countries were ranked by their total score on a scale of 0 to 37. The highest scores were achieved by the Islamic Republic of Iran, Egypt and Jordan 29, 28 and 26 respectively. Twelve of the countries (55%) scored more than half of the possible score (19). The lowest and highest scores for all countries summed were on sections related to banning smoking in public places (18) and tobacco advertising bans (66) respectively. Compliance with smoke-free policies was especially low. MPOWER programmes are accepted in the Region but there is considerable room for improvement. Input from countries based on their successes and challenges is needed to strengthen the programmes.	G. Heydari;F. Talischi; H. Algouhmani, H. A. Lando, A. E. Ahmandy. WHO MPOWER tobacco control scores in the Eastern Mediterannean countries based on the 2011 report. Eastern Mediterranean Health Journal. 2013.

Hoffman et al.	2015	Multiple	Article	Background: Government interventions are critical to addressing the global tobacco epidemic, a major public health problem that continues to deepen. We systematically synthesize research evidence on the effectiveness of government tobacco control policies promoted by the Framework Convention on Tobacco Control (FCTC), supporting the implementation of this international treaty on the tenth anniversary of it entering into force. Methods: An overview of systematic reviews was prepared through systematic searches of five electronic databases, published up to March 2014. Additional reviews were retrieved from monthly updates until August 2014, consultations with tobacco control experts and a targeted search for reviews on mass media interventions. Reviews were assessed according to predefined inclusion criteria, and ratings of methodological quality were either extracted from source databases or independently scored. Results: Of 612 reviews retrieved, 45 reviews met the inclusion criteria and 14 more were identified from monthly updates, expert consultations and a targeted search, resulting in 59 included reviews summarizing over 1150 primary studies. The 38 strong and moderate quality reviews published since 2000 were prioritized in the qualitative synthesis. Protecting people from tobacco smoke was the most strongly supported government intervention, with smoke-free policies associated with decreased smoking behaviour, secondhand smoke exposure and adverse health outcomes. Raising taxes on tobacco products also consistently demonstrated reductions in smoking behaviour. Tobacco product packaging interventions and anti-tobacco mass media campaigns may decrease smoking behaviour, with the latter likely an important part of larger multicomponent programs. Financial interventions for smoking cessation are most effective when targeted at smokers to reduce the cost of cessation products, but incentivizing quitting may be effective as well. Although the findings for bans on tobacco advertising were inconclusive, other evidence suggests they remain an important intervention. Conclusion: When designing and implementing tobacco control programs, governments should prioritize smoking bans and price increases of tobacco products followed by other interventions. Additional studies are needed on the various factors that can influence a policy's effectiveness and feasibility such as cost, local context, political barriers and implementation strategies.	S. J. Hoffman and C. Tan. Overview of systematic reviews on the health-related effects of government tobacco control policies. BMC public health. 2015;15():744.
Hopkins et al.	2010	Multiple	Article	No abstract available.	Hopkins DP, Razi S, Leeks KD, Priya Kalra G, Chattopadhyay SK, Soler RE. Smokefree policies to reduce tobacco use. A systematic review. American journal of preventive medicine. 2010;38(2 Suppl):S275-289. doi: 10.1016/j.amepre.2009.10.029
Husain et al.	2016	Multple	Article	Tobacco smoking prevalence remains low in many African countries. However, growing economies and the increased presence of multinational tobacco companies in the African Region have the potential to contribute to increasing tobacco use rates in the future. This paper used data from the 2014 Global Progress Report on implementation of theWorld Health Organization Framework Convention on Tobacco Control (WHO FCTC), aswell as the 2015 WHO report on the global tobacco epidemic, to describe the status of tobacco control and prevention efforts in countries in theWHO African Region relative to the provisions of theWHO FCTC andMPOWER package. Among the 23 countries in the African Region analyzed, there are large variations in the overall WHO FCTC implementation rates, ranging from 9% in Sierra Leone to 78% in Kenya. The analysis of MPOWER implementation status indicates that opportunities exist for the African countries to enhance compliance withWHO recommended best practices for monitoring tobacco use, protecting people fromtobacco smoke, offering help to quit tobacco use, warning about the dangers of tobacco, enforcing bans on tobacco advertising and promotion, and raising taxes on tobacco products. If tobacco control interventions are successfully implemented, African nations could avert a tobacco-related epidemic, including premature death, disability, and the associated economic, development, and societal costs.	M. J. Husain, L. M. English, N. Ramanandraibe. An overview of tobacco control and prevention policy status in Africa. Preventive Medicine, (91) S16-22, 2016.
Hyland et al.	2012	Multiple	Article	Smoke-free policies have been an important tobacco control intervention. As recently as 20 years ago, few communities required workplaces and hospitality venues to be smoke-free, but today approximately 11% of the world's population live in countries with laws that require these places to be smoke-free. This paper briefly summarises important milestones in the history of indoor smoke-free policies, the role of scientific research in facilitating their adoption, a framework for smoke-free policy evaluation and industry efforts to undermine regulations. At present, smoke-free policies centre on workplaces, restaurants and pubs. In addition, many jurisdictions are now beginning to implement policies in outdoor areas and in shared multiunit housing settings. The future of smoke-free policy development depends on credible scientific data that documents the health risks of secondhand smoke exposure. Over the next 20 years smoke-free policies will very likely extend to outdoor and private areas, and changes in the types of tobacco products that are consumed may also have implications for the nature and scope of the smoke-free policies of the future.	A. Hyland;J. Barnoya;J. E. Corral. Smoke-free air policies: past, present and future. Tob Control. 2012;21(2):154-61.
IARC et al.	2009	Multiple	Article	This publication reviews the effectiveness of measures enforced to eliminate tobacco smoking and tobacco smoke from environments where exposure takes place. It also offers a critical review of the evidence on the economic effects and health benefits (focusing on cancer, cardiovascular, and respiratory diseases) of smoke-free legislation and the adoption of voluntary smoke-free policies in households. Chapters particularly discussed the health effects of exposure and reduced exposure to second-hand tobacco smoke (SHS), evolution of smoke-free policies, economic impacts and incidental outcomes of smoke-free policies, public attitudes and compliance, effect of smoke-free policies on smoking behaviour, and home smoking restrictions.	. Evaluating the effectiveness of smoke-free policies. IARC Handbooks of Cancer Prevention, Volume 13: Tob Control. 2009;(150 cours Albert Thomas, 69372 Lyon Cedex 08, France.):.

InterAmerican Heart Foundation et al.	2010	Multiple	Report	No abstract available.	InterAmerican Heart Foundation. Framework Convention on Tobacco Control: Challenges for Latin America and the Caribbean, civil society report. Dallas, TX: InterAmerican Heart Foundation;2010.
International Union Against Tuberculosis and Lung Disease	2011	Multiple	Report	No abstract available.	Assessing compliance with smokefree laws. International Union Against Tuberculosis and Lung Disease. 2011.
International Union Against Tuberculosis and Lung Disease	2014	Multiple	Report	No abstract available.	Assessing compliance with smokefree laws, Second Edition. International Union Against Tuberculosis and Lung Disease. 2011.
Jacobson et al.	1999	Multiple	Article	No abstract available.	P. D. Jacobson;J. Wasserman. The implementation and enforcement of tobacco control laws: policy implications for activists and the industry. J of Health Politics, Policy and Law. 1999;24(3):567-598.
Jain et al.	2016	India	Article	Background: The Government of India has taken various initiatives for tobacco control by enacting comprehensive tobacco control legislation (Cigarette and Other Tobacco Products Act [COTPA], 2003). Objectives: The aim of this study was to assess the level of compliance of Sections 4, 5, 6-a, and 6-b, and 7, 8, and 9 of COTPA with respect to public places, educational institutes, point of sale (PoS), and warning on packaging (COTPA) in public places of Alwar District of Rajasthan. Methods: A cross-sectional observational study was conducted in 2014 in Alwar city and four blocks of the district. The study was done around 365 public places for observing the compliance of Section 4 of COTPA, 357 educational institutions for observing the compliance of Section 6-b of COTPA, and 357 tobacco retailers for observing the compliance of Sections 5 and 6-a of COTPA. Results: The criteria for the evaluation (the core indicators) and decision criteria for a district to qualify for the "Smoke free" status include six parameters. From the total of 365 places visited, 90% places displayed the "No-smoking" signage and out of total 328 places, 99% were as per the COTPA specification. Alwar city, Ramgarh, Thanagaji, and Alwar rural block followed the compliance of Section 4. The PoS visited Alwar district displayed 93% (332) signage and all the displayed signage followed the COTPA compliance. In Alwar city, Thanagaji, Ramgarh, and Alwar rural block, the compliance of Section 6-a was above 90%. The compliance of Section 6-b was above 90% in Alwar city, Ramgarh, Thanagaji, and Alwar rural block. Ninety-three percent (332) of the PoS did not display tobacco advertisement in Alwar district, which is a positive sign of COTPA compliance. Conclusion: This finding suggest a high level of compliance of Section 4, Section 5, Section 6-a, and Section 6-b of COTPA at Alwar district.	M. L. Jain, M. Chauhan and R. Singh. Compliance assessment of cigarette and other tobacco products act in public places of Alwar district of Rajasthan. Indian journal of public health. 2016;60(2):107-11.

Jain et al.	2014	India	Article	In 2003, the Parliament of India passed the Cigarettes and Other Tobacco Products (Prohibition of Advertisement and Regulation of Trade and Commerce, Production, Supply and Distribution) Act, 2003 (COTPA) to counter the growing societal and health burdens of tobacco in India. The major provisions (Sections 4–7) of COTPA mandate the display of pictorial health warnings on all tobacco product packets and strictly prohibit: smoking in public places, direct and indirect forms of tobacco advertisements, promotion and sponsorship of tobacco products, sale of tobacco products to a minor, and sale within 100 yards of any educational institution. However, India continues to have a high prevalence of tobacco consumption and the provisions of COTPA are routinely flouted. The purpose of the study is to analyze the trends in and the prevalence of COTPA violations in a sample test site (Haryana) to develop informed and practical tobacco control policy recommendations. Violations in five districts of the State of Haryana were observed and recorded. The data overwhelmingly show that the most fundamental provisions (Sections 4–7) of COTPA were frequently violated in each of the five districts. All districts had a high rate of noncompliance, with Sites having at least one violation of Sections 4–7 of COTPA, with rates fluctuating between nearly 70 and 90 percent. Such violations however, were unequally distributed between Sections 4–7 within the five districts with some districts having a higher frequency of Section 4 violations (Panipat and Jhajjar) and other districts with higher violation frequency of Section 5 (Mewat and Kurukshetra). However, all five districts had relatively low numbers of Section 7 violations (i.e., the required display of pictorial health warnings on all tobacco products). The study highlights the challenges of the tobacco control policy in India including: engagement of state and district level enforcement officials for effective enforcement of existing legislation, encouragement for civil society to partner and complement governmental efforts in monitoring progress and reporting violations of COTPA, and the need for supply-level controls on tobacco (e.g., pictorial health warnings and increased taxation) to reduce tobacco consumption.	Jain D, Jadav A, Rhoten K, Bassi A. The Enforcement of India's Tobacco Control Legislation in the State of Haryana: A Case Study. World Medical & Health Policy. 2014;6(4):331-346. doi: 10.1002/wmh3.116
Janghorbani et al.	2004	Iran	Article	Background - Evidence on the provision of smoke-free areas in restaurants is mainly derived from studies in industrialized countries. Evidence from industrializing countries is required for planning a well-coordinated approach to the public health problem of tobacco-related disease in these countries. Methods - To assess public opinion on tobacco control policies in restaurants, a population-based, cross-sectional, random digit-dialing telephone survey was carried out from November 2002 through January 2003. Eight-hundred and ninety-seven randomly selected subjects age 15 years or above were included in the study. Results - Ninety-four point nine percent of respondents (95% confidence interval [CI] 93.5 to 96.4) supported a total smoking ban in restaurants. Reported experiences of discomfort or symptoms from second-hand smoke in restaurants were common. The majority of respondents (60.9%; 95% CI 57.7 to 64.1) anticipated an increase in their frequency of use of restaurants after a total ban. No change in frequency of patronage was predicted by 37.9% of respondents (95% CI 34.7 to 41.1), whereas only 0.7% (95% CI 0.3 to 1.5) stated that they would dine out less often. In multivariate analyses, nonsmokers (adjusted odds ratio 3.2 [95% CI 1.3 to 7.5]) were more likely to support a total smoking ban in restaurants. Conclusion - This first comprehensive survey in Iran shows a strong community support for smoke-free dining.	M. Janghorbani;M. H. Taghdisi;E. Vingard. Public opinion on tobacco control policies in restaurants in Isfahan, Iran. Archives of Iranian Medicine. 2004;7:260-266.
John et al.	2007	India	Editorial	No abstract available.	R. M. John;S. A. Glantz. It is time to make smokefree environments work in India. Indian J of Medical Research. 2007;125(5):599-603.
Johns Hopkins Institute for Global Tobacco Control	2013	Multiple	Article	No abstract available.	Smoke-free: What does success look like? Indicators of a smoke-free jurisdiction. Johns Hopkins Bloomberg School of Public Health. 2013.

Jovicevic et al.	2011	Serbia	Article	Background: In Serbia, the new Law on Protection from Environmental Tobacco Smoke has entered into force in November 2010, banning smoking in indoor public places (health, education, child care, sports, culture, govermental institutions) and workplaces, and with moderate restrictions in pubs and restaurants.Objectives: In order to monitor and evaluate implementation and effects of the new smoke-free law, the National Committee for Tobacco Prevention of the Ministry of Health has organized the follow-up of public support, compliance, smoking cessation services activities and health benefits related to the new legislation.Data will be obtained by public surveys, from smoking cessation services, inspections and hospitals (admissions due to acute myocardial infarction).Materials and Methods: Survey of the representative sample of the Serbian population (over 1100 participants) has been carried out before the implementation and after 3 months of the implementation of the Law; it will be carried out after 6 and 12 months as well.Both rounds of survey were carried out by using the same methodology, on representative random samples of Serbian adult population (over 1000 participants each).Results: The survey revealed that the public support for the new smokefree law has increased, from 77% in the baseline study to 80% after 3 months of implementation.The majority of participants (around 90%) agreed that smoking was the significant cause of cancer, heart diseases and stroke, that ETS was harmful to nonsmokers and that smokers should take care not to expose others to tobacco smoke.In comparison with the baseline survey, the percentage of population exposed to tobacco smoke in workplaces and at schools has decreased significantly (from 45 to 35% in workplaces and from 44 to 23% at schools).The exposure to tobacco smoke in bars and restaurants haven't changed - the Law provided only minor restrictions.More than 20% of the population thinks that smoking should be completely allowed in restaurants, pubs and night clubs.The number of homes where smoking was allowed everywhere has decreased from 48% to 42%.The new law had effects on smokers as well - one quarter of smokers reduced the number of daily cigarettes and one fifth begun considering quitting smoking because of it.Among those that quit smoking, 38% said that they were motivated by this Law. More than half of the population (53%) thought that the Law was mostly or completely respected.Conclusions: Evaluation results indicate that there is a strong support for the new smoke-free law in Serbia and that, after 3 months, it is already showing positive effects.Regular follow up will enable evaluation of the Law and its impact and enable plan measures for the improvement.	A. Jovicevic;S. Krstev;N. Lazarevic;A. Dzeletovic;I. Pesic;S. Ristic. Impact of the new smoke-Free law in Serbia. European J of Cancer. 2011;47
Kalkhoran et al.	2015	Uruguay	Article	Introduction: Implementation of smokefree laws is followed by drops in hospital admissions for cardiovascular diseases and asthma. The impact of smokefree laws on use of non-hospital medical services has not been assessed. The purpose of this study is to evaluate the impact of Uruguay's national 100% smokefree legislation on non-hospital emergency care visits, hospitalizations for bronchospasm, and bronchodilator use. Methods: The monthly number of non-hospital emergency care visits and hospitalizations for bronchospasm, as well as monthly puffs of bronchodilators (total and per person), from 3 years prior to the adoption of the 100% smokefree policy on March 1, 2006, through 5 years after the policy were assessed using interrupted time series negative binomial regression. Data analysis was conducted in 2014. Results: The incidence of non-hospital emergency visits for bronchospasm decreased by 15% (incidence rate ratio [IRR]=0.85, 95% CI=0.76, 0.94) following implementation of the law. Hospitalizations for bronchospasm did not change significantly (IRR=0.89, 95% CI=0.66, 1.21). Total monthly puffs of salbutamol and ipratropium administered in the non-hospital emergency setting decreased by 224 (95% CI=-372, -76) and 179 (95% CI=-340, -18.6), respectively, from means of 1,222 and 1,007 before the law. Conclusions: Uruguay's 100% smokefree law was followed by fewer emergency visits for bronchospasm and less need for treatment, supporting adoption of such policies in low- and middle-income countries to reduce the disease burden and healthcare costs associated with smoking.	S. Kalkhoran, E. M. Sebrie, E. Sandoya and S. A. Glantz. Effect of Uruguay's National 100% Smokefree Law on Emergency Visits for Bronchospasm. American journal of preventive medicine. 2015;49(1):85-8.
Karimi et al.	2016	Kenya	Article	No abstract available.	K. J. Karimi, R. Ayah and T. Olewe. Adherence to the Tobacco Control Act, 2007: Presence of a workplace policy on tobacco use in bars and restaurants in Nairobi, Kenya. . 2016;6(9):.
Kashiwabara et al.	2011	India	Article	Smoke-free legislation is gaining popularity; however, it must accompany effective implementation to protect people from secondhand smoke (SHS) which causes 600,000 deaths annually. Increasing numbers of smoke-free cities in the world indicate that municipalities have an important role in promoting smoke-free environments. The objectives were to describe the local initiative to promote smoke-free environments and identify the key factors that contributed to the process. Observations were based on a case study on the municipal smoke-free initiatives in Chandigarh and Chennai, India. India adopted the Cigarette and Other Tobacco Products Act in 2003, the first national tobacco control law including smoke-free provisions. In an effort to enforce the Act at the local level, a civil society organization in Chandigarh initiated activities urging the city to support the implementation of the provisions of the Act which led to the initiation of city-wide law enforcement. After the smoke-free declaration of Chandigarh in 2007, Chennai also initiated a smoke-free intervention led by civil society in 2008, following the strategies used in Chandigarh. These experiences resonate with other cases in Asian cities, such as Jakarta, Davao, and Kanagawa as well as cities in other areas of the world including Mexico City, New York City, Mecca and Medina. The cases of Chandigarh and Chennai demonstrate that civil society can make a great contribution to the enforcement of smoke-free laws in cities, and that cities can learn from their peers to protect people from SHS.	M. Kashiwabara;R. Arul;H. Goswami;J. P. Narain;F. Armada. Local governments and civil society lead breakthrough for tobacco control: lessons from Chandigarh and Chennai. Indian J Public Health. 2011;55(3):234-9.

Kaufman et al.	2015	Indonesia	Article	Objective: District policies were recently put into place in Indonesia prohibiting smoking in public spaces. This study sought to (1) assess participants' general knowledge of secondhand smoke (SHS) dangers; (2) assess participants' awareness of and specific knowledge of smoke-free (SF) policies; and (3) assess the extent to which such policies are socially enforced and gather examples of successful social enforcement. Methods: Qualitative in-depth interviews and focus group discussions were conducted in Bogor and Palembang cities with both community members and key informants such as government officials, non-government agency staff, religious leaders and health workers. Results: Participants in both Palembang and Bogor find SF policy important. Although there was awareness of SHS dangers and SF policies, accurate knowledge of the dangers and an in-depth understanding of the policies varied. There was a high level of support for the SF policies in both cities among both smokers and non-smokers. Many participants did have experience asking a smoker not to smoke in an area where it was restricted, even if their comfort in doing so varied. There was, however, a higher level of comfort in telling smokers to stop or to move away from pregnant women and children. Hesitation to socially enforce the policies was especially present when asking men of status and/or community leaders to stop smoking, but overall participants felt they could comfortably ask someone to obey the law. Conclusion: Palembang and Bogor may be evolving towards creating social norms in support of prohibiting smoking in public spaces. If provided with more support from government and law officials, such as government officials themselves promoting the policies and demonstrating compliance, and renewed efforts to promote and enforce policies in general were made, Indonesians in these cities may feel more confident protecting non-smokers from SHS.	M. R. Kaufman, A. P. Merritt, R. Rimbatmaja and J. E. Cohen. 'Excuse me, sir. Please don't smoke here'. A qualitative study of social enforcement of smoke-free policies in Indonesia. Health policy and planning. 2015;30(8):995-1002.
Kaur et al.	2011	India	Article	Background: Air nicotine monitoring is an established method of measuring exposure to second hand smoke (SHS). Not much research has been done in India to measure air nicotine for the purpose of studying exposure to SHS. It is a risk factor and many diseases are known to occur among non smokers if they are exposed to second hand smoke. Objective: To conduct monitoring of air nicotine for second hand smoke exposure in public places across major cities in India. MATERIALS AND Methods: A cross sectional survey was conducted across four cities across the country, using passive air monitoring. The buildings included hospitals, secondary schools, Governmental offices, bars and restaurants. The buildings were selected through convenience sampling method keeping in view specific sentinel locations of interest. RESULT: The presence of air nicotine was recorded in most of the buildings under the study, which included government buildings, hospitals, schools, restaurants and entertainment venues (bars) in all four cities under the study. The highest median levels of air nicotine were found in entertainment venues and restaurants in cities. Conclusion: The presence of air nicotine in indoor public places indicates weak implementation of existing smoke free law in India. The findings of this study provide a baseline characterization of exposure to SHS in public places in India, which could be used to promote clean indoor air policies and programs and monitor and evaluate the progress and future smoke-free initiatives in India.	J. Kaur;V. M. Prasad. Air nicotine monitoring for second hand smoke exposure in public places in India. Indian J Community Med. 2011;36(2):98-103.
Kaushik et al.	2014	India	Article	Introduction: Cardiovascular diseases are the largest cause of mortality in India (WHO). Tobacco poses an enormous public health challenge as the most prevalent risk factor with worsening youth tobacco use patterns including increasing use among girls (GYTS India). India prohibited smoking in defined public places through legislation (COTPA) in 2003, including restaurants, to reduce youth access. Objectives: To strengthen enforcement of prohibition on smoking is public places by promoting youth engagement and ownership through enhancing youth advocacy and selfefficacy skills. Methods: 80 School and college going youth were oriented through a social behavioral intervention. Youth launched a campaign to monitor and advocate for compliance with prohibition on smoking in restaurants. The youth observed 281 randomly selected restaurants in urban states of Andhra Pradesh and Gujarat. The youth visited each restaurant thrice in one month; first to make observations on compliance status, second to orient the owners/ managers on legal provisions, and third to monitor if the restaurant complied after last visit. Crucial aspects of compliance were observed and analyzed by using McNemar's test to inform impact assessment. Results: While only 21.59% of restaurants displayed any tobacco warning at first visit, 80.68% of the restaurants had displayed a warning board. The youth also observed a six times increase in restaurants which displayed a warning board on each entrance; five times increase in restaurants displaying warning board at each floor; six times increase in restaurants displaying the board at each staircase; and while no restaurant displayed a warning board at each elevator before intervention, over 8.42% restaurants did at third visit ($p<0.001$). 83.47% restaurants displayed prescribed sized boards post intervention as against 12.81% restaurants at pre intervention. Restaurants displaying boards as specified and carry authorized person's name to report violations increased from 18.94% and 12.40% respectively to 85.06% and 81.82%. Youth categorized twice as many restaurants as 'completely smoke free' after campaign ($p<0.001$). (Figure Presented) Conclusion: Youth led smoke free restaurant campaign achieved significant impact in enhancing enforcement of smoke free public places. Youth advocates, empowered through advocacy skills building combined with ownership in targeted intervention can be powerful in improving public health.	Kaushik, U / Gupta, V. K. / Arora, M.. Youth advocacy to strengthen smoke free policy: Lessons from India. Global Heart. 2014;9(1):e56.

Kegler et al.	2014	China	Article	Background: A certain level of public support for smoke-free environments is a prerequisite for adoption and enforcement of policies and can be used as an indicator of readiness for legislative action. This study assessed support for comprehensive smoke-free policies in a range of settings such as hotels and colleges among government workers in China and identified factors associated with support for smoke-free policies. Understanding the extent to which government workers, a large segment of the working population in China, report a smoke-free workplace and support for smoke-free policies may be important indicators of readiness for strengthened policies given their role in formulating, implementing and enforcing regulations. Methods: Data were from an evaluation of the Tobacco Free Cities initiative of Emory University's Global Health Institute-China Tobacco Control Partnership. Self-administered surveys were completed by 6,646 workers in 160 government agencies in six Chinese cities. Multivariate logistic regression was used to identify factors associated with support for smoke-free worksites, bars, hotels, and colleges. Results: Over half (54.6%) of participants were male. A large percentage of the male workers smoked (45.9%,) whereas very few women did (1.9%). Fewer than 50% of government workers reported smoke-free policies at work, with 19.0% reporting that smoking is allowed anywhere. Support for smoke-free policies was generally very high, with the lowest levels of support for smoke-free bars (79.0%) and hotels (82.3%), higher levels of support for restaurants (90.0%) and worksites (93.0%), and above 95% support for hospitals, schools, colleges, public transportation and religious settings. Knowledge of the harmfulness of secondhand smoke was positively associated with support for smoke-free policies. Stricter worksite smoking policies were associated with support for smoke-free workplaces and bars, but not hotels and colleges. Women and nonsmokers were more supportive of smoke-free policies in general. Conclusion: Government workers play important roles in formulating, implementing and enforcing regulations; results suggest support for a more comprehensive approach to smoke-free environments in China among workers across a broad range of agencies.	M. C. Kegler, X. Hua, M. Solomon, Y. Wu, P. P. Zheng and M. Eriksen. Factors associated with support for smoke-free policies among government workers in Six Chinese cities: a cross-sectional study. BMC public health. 2014;14():1130.
Khan et al.	2016	Pakistan	Article	Objective: In order to limit the high prevalence of tobacco use in Pakistan various tobacco control laws have been implemented. The objective of this study is to serve as a pilot study to assess the implementation of these laws in the largest city of Pakistan, Karachi. Methods: A cross-sectional study was conducted in Karachi. The implementation of tobacco control laws in 'smoke-free' places, the adherence of tobacco companies to these laws, the regulation of cigarette sale, and the awareness and views of the general public regarding tobacco control laws were assessed via direct observation by visits and through self-administered questionnaires. Results: The implementation of tobacco control laws in 'smoke-free' public places was found to be poor. Out of 37, only 23(62%) brands displayed pictorial warnings on their packs. 3(8%) of the brands were available in two different kinds of packs, both with and without pictorial warnings. Cigarette sale to minors was taking place at 80(85%) of the visited cigarette outlets. 50(53%) of the outlets displayed cigarette advertisements in the form of posters. 46(40%) of the persons questioned had awareness regarding the existence of ban on smoking in public places and 126(90%) of these were in favour of it. Conclusions: The implementation of tobacco control law in Pakistan is poor. Non adherence to the law in public places was alarmingly high. Also, the study demonstrates the poor compliance to the tobacco control laws by tobacco companies. The sale of cigarettes is almost unregulated.	J. A. Khan, A. M. Amir Humza Sohail and M. A. Arif Maan. Tobacco control laws in Pakistan and their implementation: A pilot study in Karachi. JPMA. The Journal of the Pakistan Medical Association. 2016;66(7):875-9.
Kosir et al.	2009	Multiple	Report	No abstract available.	Kosir M, Gutierrez K, Lessons Learned Globally: Secondhand Smoke Mass Media Campaigns. Saint Paul, Minnesota, United States: Global Dialogue for Effective Stop Smoking Campaigns; 2009.
Kumar et al.	2014	India	Article	Background: India has been implementing smoke-free legislation since 2008 prohibiting smoking in public places. This study aimed to assess the level of compliance with smoke-free legislation (defined as the presence of no-smoking signage and the absence of active smoking, smoking aids, cigarette butts/bidi ends and smoking smell) and the role of enforcement systems in Indian jurisdictions. Methods: This was a cross-sectional, retrospective review of reports and primary data sheets of surveys conducted in 38 selected jurisdictions across India in 2012-2013. Results: Of 20 455 public places (in 38 jurisdictions), 10 377 (51%) demonstrated full compliance with smoke-free law. Educational institutions and healthcare facilities performed well at 65% and 62%, respectively, while eateries and frequently visited other public places (such as bus stands, railway stations, shopping malls, stadia, cinema halls etc.) performed poorly at 37% and 27%, respectively. Absence of no-smoking signage was the largest contributor to non-compliance across all types of public places. Enforcement systems were present in all jurisdictions, but no associations could be demonstrated between these and smoke-free compliance. Conclusion: Smoke-free compliance in public places in India was suboptimal and was mainly related to the absence of no-smoking signage. This warrants further pragmatic and innovative ways to improve the situation.	R. Kumar;S. Goel;A. D. Harries;P. Lal;R. J. Singh;A. M. Kumar;N. C. Wilson. How good is compliance with smoke-free legislation in India? Results of 38 subnational surveys. Int Health. 2014;():.

Kyaing et al.	2011	Multiple	Article	This paper examines the social, cultural, economic and legal dimensions of tobacco control in the South-East Asia Region in a holistic view through the review of findings from various studies on prevalence, tobacco economics, poverty alleviation, women and tobacco and tobacco control laws and regulations. Methods were Literature review of peer reviewed publications, country reports, WHO publications, and reports of national and international meetings on tobacco and findings from national level surveys and studies. Tobacco use has been a social and cultural part of the people of South-East Asia Region. Survey findings show that 30% to 60% of men and 1.8% to 15.6% of women in the Region use one or the other forms of tobacco products. The complex nature of tobacco use with both smoking and smokeless forms is a major challenge for implementing tobacco control measures. Prevalence of tobacco use is high among the poor and the illiterate. It is higher among males than females but studies show a rising trend among girls and women due to intensive marketing of tobacco products by the tobacco industry. Tobacco users spend a huge percent of their income on tobacco which deprives them and their families of proper nutrition, good education and health care. Some studies of the Region show that cost of treatment of diseases attributable to tobacco use was more than double the revenue that governments received from tobacco taxation. Another challenge the Region faces is the application of uniform tax to all forms of tobacco, which will reduce not only the availability of tobacco products in the market but also control people switching over to cheaper tobacco products. Ten out of eleven countries are Parties to the WHO Framework Convention on Tobacco Control and nine countries have tobacco control legislation. Enforcement of control measures is weak, particularly in areas such as smoke-free environments, advertisement at the point of sale and sale of tobacco to minors. Socio-cultural acceptance of tobacco use is still a major challenge in tobacco control efforts for the governments and stakeholders in the South-East Asia Region. The myth that chewing tobacco is less harmful than smoking tobacco needs to be addressed with public awareness campaigns. Advocacy on the integration of tobacco control with poverty alleviation campaigns and development programs is urgently required. Law enforcement is a critical area to be strengthened and supported by WHO and the civil society organizations working in the area of tobacco control.	N. N. Kyaing;M. A. Islam;D. N. Sinha;S. Rinchen. Social, economic and legal dimensions of tobacco and its control in South-East Asia region. Indian J Public Health. 2011;55(3):161-8.
Lambert et al.	2013	Seychelles	Report	No abstract available.	Lambert P, Donley K. Best practices in implementation of Article 8 of the WHO FCTC case study: Seychelles. Geneva: World Health Organization;July 2013.
Lambert et al.	2013	South Africa	Report	No abstract available.	Lambert P, Donley K. Best practices in implementation of Article 8 of the WHO FCTC case study: South Africa. Geneva: World Health Organization;July 2013.
Lazuras et al.	2012	Multiple	Article	Objective: Identify the psychosocial variables that predict smokers' compliance with smoke-free policies at work, and non-smokers' assertiveness for smoke-free rights in Greek and Bulgarian workplaces. Methods: Data were collected from employees in Greece and Bulgaria. The main outcome measures were smokers' compliance with smoke-free policies, and non-smokers' assertiveness intentions. Demographic variables, tobacco use and dependence, as well as beliefs about second-hand smoke (SHS) exposure and smoking at work were also assessed. Results: Regression analyses showed that smokers' compliance with smoke-free policies was predicted by age, perceived health risks of smoking, and beliefs related to the benefits of smoking at work. Non-smokers' assertiveness was predicted by annoyance from exposure to SHS at work, and assertiveness-related social cognitions (e.g., attitudes, social norms, and self-efficacy). Conclusions: Interventions to promote support for tobacco control policies at work in Greece and Bulgaria may benefit from targeting smokers' beliefs about the actual effects of tobacco use on health and job performance. Accordingly, efforts to promote non-smokers assertiveness should build stronger assertiveness-related attitudes, convey anti-smoking normative messages, and strengthen self-efficacy skills.	L. Lazuras;M. Zlatev;A. Rodafinos;J. R. Eiser. Smokers' compliance with smoke-free policies, and non-smokers' assertiveness for smoke-free air in the workplace: a study from the Balkans. Int J Public Health. 2012;57(5):769-75.
Lee et al.	2012	Multiple	Article	Purpose: To understand transnational tobacco companies' (TTCs) practices in low and middle-income countries which serve to block tobacco-control policies and promote tobacco use. Methods: Systematic review of published research on tobacco industry activities to promote tobacco use and oppose tobacco-control policies in low and middle-income countries. Results: TTCs' strategies used in low and middle-income countries followed four main themes-economic activity; marketing/promotion; political activity; and deceptive/manipulative activity. Economic activity, including foreign investment and smuggling, was used to enter new markets. Political activities included lobbying, offering voluntary self-regulatory codes, and mounting corporate social responsibility campaigns. Deceptive activities included manipulation of science and use of third-party allies to oppose smoke-free policies, delay other tobacco-control policies, and maintain support of policymakers and the public for a pro-tobacco industry policy environment. TTCs used tactics for marketing, advertising, and promoting their brands that were tailored to specific market environments. These activities included direct and indirect tactis, targeting particular populations, and introducing new tobacco products designed to limit marketing restrictions and taxes, maintain the social acceptability of tobacco use, and counter tobacco-control efforts. Conclusions: TTCs have used similar strategies in high-income countries as these being described in low and middle-income countries. As required by FCTC Article 5.3, to counter tobacco industry pressures and to implement effective tobacco-control policies, governments and health professionals in low and middle-income countries should fully understand TTCs practices and counter them. (copyright) 2012 Springer Science+Business Media B.V.	S. Lee;P. M. Ling;S. A. Glantz. The vector of the tobacco epidemic: tobacco industry practices in low and middle-income countries. Cancer Causes and Control. 2012;.

Leischow et al.	2013	Multiple	Article	No abstract available.	Leischow SJ, Ayo-Yusuf O, Backinger CL. Converging research needs across framework convention on tobacco control articles: making research relevant to global tobacco control practice and policy. Nicotine & tobacco research. 2013;15(4):761-766. doi: 10.1093/ntr/nts199
Levy et al.	2003	Multiple	Article	Our objective was to review the research on the effects of public clean air laws on smoking rates, compare these effects to those found in studies on the impact of private worksite restrictions and derive estimates of the potential reductions in smoking rates that might be expected from the implementation of the two types of policies. Data sources were computerized databases, references identified from pertinent peer-reviewed journal articles and books, and suggestions by experts on tobacco control policy. Comprehensive public clean air laws have the potential to reduce prevalence and consumption rates of the entire population (including non-working and non-indoor working smokers) by about 10%. Studies on private worksite regulations also suggest that strong worksite restrictions have the potential to reduce the prevalence rate of the entire population by about 6% over the long-term and the quantity smoked by continuing smokers by 2-8%, depending on the length of time after the ban. Further research is needed on the effects of the different types of public clean air policies on the entire smoking population and on different sociodemographic groups, how the effects of public clean indoor air laws depend on private restrictions already in place, and how the effect of private restrictions depend on whether or not they are supported by public clean air laws.	D. T. Levy;K. B. Friend. The effects of clean indoor air laws: What do we know and what do we need to know?. Health Education Research. 2003;18(5):592-609.
Levy et al.	2001	Multiple	Article	No abstract available.	Levy DT, Friend K. A framework for evaluating and improving clean indoor air laws. J Public Health Manag Pract. 2001;7(5):87-96.
Li et al.	2013	China	Article	Background:The Shanghai Public Places Smoking Control Legislation was implemented in March 2010 as the first provincial-level legislation promoting smoke-free public places in China.Objective:To evaluate the compliance with this policy as well as its impact on exposure to secondhand smoke (SHS), respiratory symptoms, and related attitudes among employees in five kinds of workplaces (schools, kindergartens, hospitals, hotels, and shopping malls). Methods:A cross-sectional survey was conducted six months before and then six months after the policy was implemented. Five types of occupational employees from 52 work settings were surveyed anonymously using multistage stratified cluster sampling.Results:Six months after implementation, 82% of the participants agreed that "legislation is enforced most of the time". The percentage of self-reported exposure to secondhand smoke declined from round up to 49% to 36%. High compliance rates were achieved in schools and kindergartens (above 90%), with less compliance in hotels and shopping malls (about 70%). Accordingly, prevalence of exposure to SHS was low in schools and kindergartens (less than 10%) and high in hotels and shopping malls (40% and above). The prevalence of respiratory and sensory symptoms (e.g., red or irritated eyes) among employees decreased from 83% to 67%. Conclusions:Initial positive effects were achieved after the implementation of Shanghai Smoking Control legislation including decreased exposure to SHS. However, compliance with the policies was a considerable problem in some settings. Further evaluation of such policy implementation should be conducted to inform strategies for increasing compliance in the future. (copyright) 2013 Li et al.	X. Li;J. Gao;Z. Zhang;M. Wei;P. Zheng;E. J. Nehl;F. Y. Wong;C. J. Berg. Lessons from an Evaluation of a Provincial-Level Smoking Control Policy in Shanghai, China. PLoS ONE. 2013;8(9):.
Li et al.	2010	China	Article	Objective: To examine levels of support for comprehensive smoke-free policies in six large Chinese cities. Methods: Data from Wave 1 of the International Tobacco Control (ITC) China Survey (April-August 2006) were analysed. The ITC China Survey employed a multistage sampling design in Beijing, Shenyang, Shanghai, Changsha, Guangzhou and Yinchuan (none of which has comprehensive smoke-free policies in place). Face-to-face interviews were conducted with 4815 smokers and 1270 non-smokers. Multivariate logistic regression models were used to identify factors associated with support for comprehensive smoke-free policies. Results: About one in two Chinese urban smokers and four in five non-smokers believed that secondhand smoke (SHS) causes lung cancer. The majority of respondents supported comprehensive smoke-free policies in hospitals, schools and public transport vehicles while support for smoke-free workplaces, restaurants and bars was lower. Levels of support were generally comparable between smokers and non-smokers. Support for comprehensive smoke-free policies was positively associated with knowledge about the harm of SHS. Respondents who worked in a smoke-free worksite or who frequented smoke-free indoor entertainment places were more likely to support comprehensive smoking restriction in bars and restaurants. Conclusion: Considerable support for smoke-free policies exists in these six large cities in China. Greater public education about the dangers of SHS may further increase support. Experiencing the benefits of smoke-free indoor entertainment places and/or workplaces increases support for these policies and suggests that some initial smoke-free policy implementation may hasten the diffusion of these public health policies.	Q. Li;A. Hyland;R. O'Connor;G. Zhao;L. Du;X. Li;G. T. Fong. Support for smoke-free policies among smokers and non-smokers in six cities in China: ITC China Survey.

Liu et al.	2014	China	Article	Field observation of patron smoking behaviors and multiple sampling approaches were conducted in 79 restaurants and bars in Beijing, 2010, two years after implementing the governmental smoking regulations. Smoking was observed in 30 visits to 22 of the 37 nominal nonsmoking venues during peak-patronage times and six visits to four of the 14 nominal nonsmoking sections. The median area SHS concentrations during peak-patronage time were 27, 15, 43 and 40 mug/m3 in nominal nonsmoking venues, nonsmoking sections, smoking sections, and smoking venues, respectively, as indicated by the difference between indoor and outdoor PM2.5 levels; and 1.4, 0.6, 1.7 and 2.7 mug/m3 , respectively, as indicated by airborne nicotine. In the 27 venues with sampling of different approaches and over different time periods, the median nicotine concentration was 1.8 mug/m3 by one-hour peak-patronage-time sampling, 1.1 mug/m3 by one-day active area sampling, 2.5 mug/m3 by one-day personal sampling, and 2.3 mug/m3 by week-long passive sampling. No significant differences of nicotine levels were observed among venues/sections with different nominal smoking policies by all sampling approaches except during peak-patronage time. This study showed that the 2008 Beijing governmental smoking restriction has been poorly implemented, and SHS exposures in Beijing restaurants and bars remain high. This article is protected by copyright. All rights reserved.	R. Liu;Y. Jiang;Q. Li;S. K. Hammond. Assessing Exposure to Secondhand Smoke in Restaurants and Bars Two Years After the smoking Regulations in Beijing, China. Indoor Air. 2014;():.
Liu et al.	2014	China	Article	Introduction: In 2006, the World Health Organization Framework Convention on Tobacco Control became effective in mainland China. In 2007, advocacy on voluntary smoking bans in restaurants was initiated in Beijing, and in 2008 the Beijing government implemented a smoking regulation, requiring big restaurants to prohibit or restrict smoking. Objectives: To evaluate the efficacy of different smoking policies adopted by Beijing restaurants and bars from 2006 to 2010. Methods: The study conducted field observations of patron smoking behavior and monitored fine particulate matter from secondhand smoke (SHS PM) from 91, 85, 94 and 79 Beijing restaurants and bars in 2006, 2007, 2008 and 2010, respectively, during peak-patronage times, with overlaps of venues during each two years. Area nicotine sampling during peak patronage times and servers' personal nicotine sampling during their working shifts were also conducted in 2010. Results: Smoking was nominally prohibited or restricted in 18% of restaurants and bars monitored in 2006, in 11% of venues in 2007, in 83% of venues in 2008, and in 69% of venues in 2010. However, smoking was observed in more than 40% of the nominal nonsmoking venues/sections in 2008 and 2010. The median of observed patron active smoker density (ASD) was 0.24, 0.27, 0.00 and 0.10 active smokers per 100 m3 in 2006, 2007, 2008 and 2010, respectively. The median of SHS PM concentrations was 53, 83, 18 and 27 mug/m3, respectively. In 2010, both the median SHS PM and air nicotine concentrations in designated nonsmoking sections were about 40% of those in designated smoking sections, according to simultaneous sampling in both sections. Servers' personal exposure to air nicotine was quite similar in venues with different nominal smoking policies. In the 15 venues followed from 2006 to 2010, SHS PM concentrations changed randomly from 2006 to 2007, decreased in most venues in 2008, and then increased to some extent in 2010. Conclusion: Voluntary smoking policy is rarely adopted and cannot protect people from SHS exposure in restaurants and bars. The 2008 Beijing governmental smoking regulation failed to significantly reduce SHS exposure shortly or two years after its implementation. Restricting smoking to designated sections cannot eliminate SHS exposure.	R. Liu;Y. Jiang;M. J. Travers;Q. Li;S. K. Hammond. Evaluating the efficacy of different smoking policies in restaurants and bars in Beijing, China: a four-year follow-up study. Int J Hyg Environ Health. 2014;217(1):41649.
Liu et al.	2011	China	Article	The China Global Adults Smoking Survey, which was done by the China Center for Disease Control and Prevention, reported that 53% of men aged 15 years and above are smokers; about the same number as in 2002. Furthermore, it is estimated that 72% of non-smokers, including 180 million children, are exposed to secondhand smoke. There are several reasons for China's lack of progress in tobacco control, including weak support from the government and strong resistance from the tobacco industry. In 2009, the Chinese Ministry of Health issued the Decision on Banning Smoking in All Health Facilities by 2011, requiring that 50% of the health facilities in China should be smoke-free by the end of 2010. In China, a governmental decision with no statutory power might not be vigorously implemented. In a bid to accelerate progress, the Chinese Ministry of Health's Smoke-Free Health Facilities Campaign Office announced in May, 2010 that the Ministry itself would launch a campaign to ban smoking in all their offices and in all Ministry-supervised health facilities. (PsycINFO Database Record (c) 2012 APA, all rights reserved)	Yuanli Liu;Lincoln Chen. New medical data and leadership on tobacco control in China. The Lancet. 2011;377(9773):1218-1220.
Lopez et al.	2011	Mexico	Article	No abstract available.	Lopez CM, Ruiz JA, Shigematsu LM, Waters HR. The economic impact of Mexico City's smoke-free law. Tobacco control. 2011;20(4):273-278. doi: 10.1136/tc.2010.036467
Luo et al.	2015	China	Article	Despite the perceived success of educational campaigns and smoking bans in public places in China, the actual effects have not been investigated. This study examines the effects of the two policies by major characteristics of smokers and whether the affected smokers have intention to quit smoking. A cross-sectional survey was conducted in 17 cities in China and 16,616 participants were selected using multistage stratified sampling. Logistic regression models were used to examine the effects of educational campaigns and smoking bans in public places on their intention to quit smoking. Results show that the Chinese government should try every means to build its tobacco control publicity and implement various forms of public educational campaigns to enhance smokers' knowledge of the health consequences of smoking. In addition, China should emphasize the enforcement of the existing smoking prohibitions and regulations by implementing local tobacco control legislation and total prohibitions in all public places and workplaces.	B. Luo, L. Wan, L. Liang and T. Li. The effects of educational campaigns and smoking bans in public places on smokers' intention to quit smoking: Findings from 17 cities in China. . 2015;2015():.

Lv et al.	2011	China	Article	As per China's ratification of the WHO Framework Convention on Tobacco Control (FCTC), it should have implemented effective packaging and labelling measures prior to 9 January 2009 and enacted a comprehensive ban on all tobacco advertising, promotion and sponsorship prior to 9 January 2011. In addition, universal protection against secondhand tobacco smoke should have been implemented before 9 January 2011 by ensuring that all indoor workplaces, all indoor public places, all public transportation and possibly other (outdoor or quasi-outdoor) public places are free of secondhand smoke. The authors conducted a review of various sources of information to determine the current status of FCTC implementation in mainland China. Even though China has made considerable efforts to implement the FCTC, there is still a significant gap between the current state of affairs in China and the requirements of the FCTC. The Chinese tobacco monopoly under which commercial and other vested interests of the tobacco industry are jeopardizing tobacco control efforts is thought to be the most crucial obstacle to the effective implementation of the FCTC across the country.	J. Lv;M. Su;Z. Hong;T. Zhang;X. Huang;B. Wang;L. Li. Implementation of the WHO Framework Convention on Tobacco Control in mainland China. Tob Control. 2011;20(4):309-14.
Mackay	2013	Multiple	Article	No abstract available.	Mackay J. The role of research on the development and implementation of policy. Nicotine Tob Res. 2013;15(4):757-760. doi: 10.1093/ntr/nts197
Madanat et al.	2009	Jordan	Article	As one of the first countries to ratify the WHO Framework Convention on Tobacco Control, Jordan has signaled an interest in stronger anti-tobacco restrictions. This study examines whether Jordanian students believe smoking is an individual right or a social issue, and if they would support more stringent policies and enforcement. Undergraduate Jordanian students (n = 1211) from public and private universities completed the survey. Never smokers scored significantly higher on smoking being a social issue that required public policy response (p-value < .001); whereas smokers scored significantly higher with all individual right items. Ample opportunity exists for developing and enforcing stronger tobacco policies both on college campus and generally in the country. However, increasing tobacco taxes may need to be preceded by health communication campaigns that increase knowledge of the effectiveness of the tobacco tax in reducing use and resultant premature deaths.	H. N. Madanat;M. D. Barnes;E. C. Cole;R. Njord. Determining responsibility for smoking in society: accountability continuum for Jordanian college students and its implication on FCTC implementation. Int Q Community Health Educ. 2009;30(3):239-55.
Mathers et al.	2006	Multiple	Article	No abstract available.	Mathers CD, Loncar D. Projections of global mortality and burden of disease from 2002 to 2030. PLoS Med. 2006;3(11):e442. doi: 10.1371/journal.pmed.0030442
Mendis et al.	2011	Multiple	Article	No abstract available.	Mendis S, Alwan A, eds. Prioritized research agenda for prevention and control of noncommunicable diseases, Geneva, World Health Organization, 2011.
Mendis et al.	2011	Multiple	Article	No abstract available.	Mendis S, Alwan A, eds. Prioritized research agenda for prevention and control of noncommunicable diseases, Appendix. Geneva, World Health Organization, 2011.
Minh et al.	2016	Vietnam	Article	In Vietnam, the WHO Framework Convention on Tobacco Control (WHO FCTC) took effect in March 2005 while MPOWER has been implemented since 2008. This paper describes the progress and challenges of implementation of the MPOWER package in Vietnam. We can report that, in term of monitoring, Vietnam is very active in the Global Tobacco Surveillance System, completing two rounds of the Global Adult Tobacco Survey (GATS) and three rounds of the Global Youth Tobacco Survey (GYTS). To protect people from tobacco smoke, Vietnam has issued and enforced a law requiring comprehensive smoking bans at workplaces and public places since 2013. Tobacco advertising and promotion are also prohibited with the exception of points of sale displays of tobacco products. Violations come in the form of promotion girls, corporate social responsibility activities from tobacco manufacturers and packages displayed by retail vendors. Vietnam is one of the 77 countries that require pictorial health warnings to be printed on cigarette packages to warn about the danger of tobacco and the warnings have been implemented effectively. Cigarette tax is 70% of factory price which is equal to less than 45% of retail price and much lower than the recommendation of WHO. However, Vietnam is one of the very few countries that require manufacturers and importers to make "compulsory contributions" at 1-2% of the factory price of cigarettes sold in Vietnam for the establishment of a Tobacco Control Fund (TCF). The TCF is being operated well. In 2015, 67 units of 63 provinces/cities, 22 ministries and political-social organizations and 6 hospitals received funding from TCF to implement a wide range of tobacco control activities. Cessation services have been starting with a a toll-free quit-line but need to be further strengthened. In conclusion, Vietnam has constantly put efforts into the tobacco control field with high commitment from the government, scientists and activists. Though several remarkable achievements have been gained, many challenges remain. To overcome those challenges, implementation strategies that take into account the contextual factors and social determinants of tobacco use in Vietnam are needed.	H. V. Minh, T. T. Ngan, V. Q. Mai, N. T. My, H. Chung le, V. D. Kien, T. T. Anh, N. B. Ngoc, V. V. Giap, N. M. Cuong, P. D. Manh and K. B. Giang. Tobacco Control Policies in Vietnam: Review on MPOWER Implementation Progress and Challenges. Asian Pacific journal of cancer prevention : APJCP. 2016;17 Suppl():42744.

Mohamed et al.	2011	Multiple	Report	No abstract available.	Mohamed MK. WHO smoke-free city case study: Tobacco-free cities for smoke-free air: A case study in Mecca and Medina. Geneva: World Health Organization;2011.
Mohlman et al.	2013	Egypt	Article	Introduction: Tobacco use in low- to middle-income countries is a major public health concern for both smokers and those exposed to environmental tobacco smoke (ETS). Egypt has made important strides in controlling tobacco use, but smoking and ETS remain highly prevalent. This randomized intervention sought to improve the target population's knowledge regarding the hazards of smoking and ETS and to change attitudes and smoking behaviors within the community and the household. Methods: In this 2005-2006 study in Egypt's Qalyubia governorate, trained professionals visited schools, households, mosques, and health care centers in rural villages randomly selected for the intervention to discuss the adverse effects of smoking and ETS exposure and ways to reduce one's ETS exposure. Data collected in interviewer-facilitated surveys before and after the intervention period were analyzed in pairwise comparisons with data from control villages to assess the effectiveness of the intervention in achieving its aims. Results: The intervention group showed a greater increase in understanding the dangers associated with smoking cigarettes and waterpipes and became more proactive in limiting ETS exposure by asking smokers to stop, avoiding areas with ETS, and enacting smoking bans in the home. However, the intervention had little to no impact on the number of smokers and the amount of tobacco smoked. Conclusions: Results are consistent with previous studies showing that changing smokers' behavior can be difficult, but community-wide efforts to reduce ETS exposure through smoking bans, education, and empowering people to ask smokers to stop are effective. The method can be generalized to other settings.	M. K. Mohlman;D. N. K. Boulos;M. El-Setouhy;G. Radwan;K. Makambi;I. Jillson;C. A. Loffredo. A randomized, controlled community-wide intervention to reduce environmental tobacco smoke exposure. Nicotine & Tobacco Research. 2013;15:1372-1381.
Movsisyan et al.	2014	Armenia	Article	Objectives: This study aimed to measure the 5-year progress in the implementation of WHO Framework Convention on Tobacco Control (FCTC) in Armenia by applying the Tobacco Control Scale, a rapid assessment tool developed to assess the strength of tobacco control policies in Europe. Setting: Armenia, an economy in transition, has extreme smoking rates among men (62.5%) despite acceding to FCTC in 2004. However, little research has been carried out to evaluate Armenia's progress in tobacco control. Methods: The Tobacco Control Scale total score was estimated for Armenia using the original methodology; however, a different source of data was used in estimating the subscores on tobacco price and tobacco control spending. Results: Armenia's total score on Tobacco Control Scale has considerably improved from 2005 to 2009, mostly due to larger health warnings and advertising ban, and increased public spending on tobacco control. The scores for smoke-free public places, advertising ban, health warnings and treatment categories were below the European average in 2005 and 2007, while the price score was higher. Neither total tobacco control score nor any of its components showed a significant predictive value in a simple regression analysis using the total score and subscores as predictors for log-transformed per capita tobacco consumption. Conclusions: Higher than the European average price score for Armenia cannot be explained by the concept of affordability alone and may reflect a measurement error due to peculiarities of transition economies. The applicability of the Tobacco Control Scale could be limited to countries with mature economies, but not to transition countries such as Armenia with different social, political and economic environment. The scale modification, such as an adjustment for the policy enforcement and the effectiveness of public tobacco control spending along with alternative measures of affordability would be warranted to enhance its applicability in low-income and middle-income countries.	N. K. Movsisyan;G. N. Connolly. Measuring Armenia's progress on the Tobacco Control Scale: An evaluation of tobacco control in an economy in transition, 2005-2009. BMJ Open. 2014;4(2):.
Nakkash et al.	2010	Lebanon	Article	No abstract available.	Nakkash RT, Khalil J, Chaaya M, Afifi RA. Building research evidence for policy advocacy: a qualitative evaluation of existing smoke-free policies in Lebanon. Asia-Pacific Journal of Public Health. 2010;22(3 Suppl):168S-174S. doi: 10.1177/1010539510373020

Nemakhavhani and H et al.	2016	South Africa	Article	Background: The Tobacco Products Control Act, No. 83 of 1993 was introduced in South Africa in 1993. Due to the shortfalls of the 1993 Act, the Tobacco Products Control Amendment Act, No. 12 of 1999 was passed in 1999. The regulation relating to smoking of tobacco products in public places was gazetted in 2000 and implemented in 2001. SETTING: The setting for the study was all selected registered licensed bar-lounges including restaurants within the municipality. PURPOSE OF THE STUDY: To examine compliance levels with the current smoke-free regulation at bar-lounges and restaurants in Thulamela Municipality. Objectives OF THE STUDY: To observe whether structural alterations had been effected to accommodate smoking patrons. To observe whether tobacco-related litter was present in nonsmoking areas and in the outdoor areas within 5 meters of windows and entry ways. To observe whether individuals or groups engaged in smoking in non-smoking areas. Methods: An observational survey was conducted to measure the level of compliance by lounges and restaurants in Thulamela Municipality. A convenience sampling method was used to sample 56 bar-lounges, including restaurants. Data were collected using an observation log. Collected data were analysed using SPSS 20.0. Results: The study findings noted low compliance with the legislation with only one establishment (1.8%) complying with the requirements of the legislation. Conclusion: The level of compliance with the legislation is in a very low state in Thulamela Municipality. Further research is needed to explore factors influencing non-compliance with the regulation.	T. R. Nemakhavhani and H. A. Akinsola. Survey of bar-lounges and restaurants regarding compliance with the current smoke-free regulation in Thulamela Municipality, South Africa. African journal of primary health care & family medicine. 2016;8(2):e1-6.
Obeidat et al.	2016	Multiple	Article	Several Eastern Mediterranean (EM) countries, including Jordan, suffer from high smoking prevalence but weak implementation of smoking bans (SB). Public support (PS) influences successful implementation of SB, but little is known about PS for SB in EM countries with weak SB implementation. We conducted a cross-sectional survey measuring knowledge and perceptions of a large purposive sample of the Jordanian public regarding tobacco harms and anti-tobacco laws. Among 1169 respondents, 46% of whom used tobacco, PS for SB varied from 98% to 39% based on venue, being highest for health facilities and lowest for coffee shops. In venues with relatively lower PS (restaurants, coffee shops), lower educational groups, older age groups, nonsmokers, and those who had more knowledge regarding tobacco and secondhand smoke harms were significantly more likely to support SB than the highest educational group, the youngest age group, smokers, and those who had less knowledge (respectively). Our results suggest that aggressive promotion of SB is needed in countries like Jordan (where smoking is increasing), tailored to venue and specific sociodemographic characteristics of the public accessing these venues, particularly restaurants and coffee shops. Multifaceted health messages that enhance public knowledge can be of benefit in improving PS for SB.	N. A. Obeidat, H. S. Ayub, R. K. Bader, A. S. Shtaiwi, R. A. Shihab, M. A. Habashneh and F. I. Hawari. Public support for smoke-free policies in Jordan, a high tobacco burden country with weak implementation of policies: Status, opportunities, and challenges. . 2016;11(10):1246-1258.
Oberg et al.	2011	Multiple	Article	No abstract available.	Oberg M, Jaakkola MS, Woodward A, Peruga A, Pruss-Ustun A. Worldwide burden of disease from exposure to second-hand smoke: a retrospective analysis of data from 192 countries. Lancet. 2011;377:139-146. doi: 10.1016/S0140-6736(10)61388-8
Odhiambo et al.	2011	Kenya	Report	No abstract available.	Odhiambo F, Mutai J, Endau W, Anaya L, Matete V, Nato J. WHO smoke-free city case study: Smoke-free Nakuru – the first east African city implementing subnational smoke-free policy. Geneva: World Health Organization;2011.
Olasky et al.	2012	Multiple	Article	Second hand tobacco smoke (SHS) is an environmental toxin and an established cause of cardiovascular disease in nonsmokers. Smoke free laws reduce SHS and its downstream cardiovascular disease, but until recently evidence to support smoke free law implementation in low and middle income country settings was limited. In 14 low and middle income nations surveyed by the Global Adult Tobacco Survey active smoking prevalence in adults (>/=15 years old) was universally higher in males (range 21.6-60.2%) compared with females (0.5-24.4%), and the highest burden of SHS exposure was in women (strong positive association between male/female active smoking ratio and female SHS exposure prevalence). A systematic review was conducted of MEDLINE-indexed studies of self-reported SHS exposure and cardiovascular harms in low or middle income nations. Eight papers reported the association of SHS with ischemic heart disease and four reported the association of SHS with stroke. For all the studies, and almost all sources of SHS surveyed, a strong positive association between SHS and ischemic heart disease (main relative odds ratio range 1.17-2.36) and SHS and stroke (odds ratio or hazard ratio 1.41-1.49). Prevalence of SHS exposure is high in low and middle income nations, especially among women. Epidemiologic evidence supports the conclusion that SHS harms are the same across low, middle and high income nations. Governments have an obligation to protect citizens from SHS exposure, enforcing smoke-free legislation and providing public education about SHS harms.	S. J. Olasky;D. Levy;A. Moran. Second hand smoke and cardiovascular disease in Low and Middle Income Countries: a case for action. Global Heart. 2012;7(2):151-160 e5.

Olowookere et al.	2014	Nigeria	Article	Objective. This study determined the awareness and attitude towards the Osun state prohibition of smoking in public places law. Method. Descriptive cross-sectional study design. 520 consenting respondents recruited using a convenience sampling method were interviewed using a semi-structured questionnaire covering their smoking pattern, awareness and attitude towards the law of prohibition of smoking in public places in Osun State. Data analyzed using descriptive and chi-square statistics. Results: Only 38% were aware of the law while none had seen the document. Fifty six percent felt cigarette smoking is a problem that required the law to be implemented, while only 20% agreed that the law will stop tobacco use. The radio (58%), bill boards (45%) and newspapers (44%) were the major sources of awareness of the law. The perception of risk posed to the public and family health by cigarette smoking was poor among the participants. Conclusion: There is poor awareness and attitude to the law of prohibition of smoking in public places in Osun State. It is necessary to increase sensitization of the general public and enforcement of the law. (copyright) 2014 Olowookere et al.; licensee BioMed Central Ltd.	S. A. Olowookere;E. G. Adepoju;O. O. Gbolahan. Awareness and attitude to the law banning smoking in public places in Osun State, Nigeria. Tobacco Induced Diseases. 2014;12(1):.
Onigbogi et al.	2015	Nigeria	Article	Background: One of the requirements of the Osun State smoke-free legislation is to ensure smoke-free enclosed and partially enclosed workplaces. This survey was conducted to assess the knowledge and attitude of workers in indoor bars, beer parlors and discotheques to smoke-free legislation in general and the Osun State smoke-free law in particular. Methods: A convenience sampling of 36 hospitality centers was conducted. Interviewer-administered questionnaires were used to elicit responses about the objectives from non-smoking workers. The questionnaires had sections on knowledge of the Osun State smoke-free law, attitude toward the law and smoke-free legislation in general and exposure to second-hand tobacco smoke by the workers. Questions were also asked about the second-hand tobacco smoking status of these workers. The data were analyzed using SPSS version 15.0. Results: We had 154 participants recruited into the study. There were 75 males (48.0%) and 79 females (52.0%). On the overall, respondents had a good knowledge of the effects of second-hand smoke on health (70.2%) with 75.0% of them being aware of the general smoke-free law and 67.3% being aware of the Osun State smoke-free law although none of them had ever seen a copy of the law. A high proportion (60.0%) was in support of the Osun smoke-free law although all of them think that the implementation of the law could reduce patronage and jeopardize their income. Attitude toward second-hand smoking was generally positive with 72.0% of them having no tolerance for second-hand tobacco smoke in their homes. Most participants (95.5%) had been exposed to tobacco smoke in the workplace within the past week. Conclusion: Despite the high level of awareness of the respondents about the dangers of second hand smoke and their positive attitude to smoke-free laws, nearly all were constantly being exposed to second hand smoke at work. This calls for policy level interventions to improve the implementation of the smoke-free law.	O. O. Onigbogi, O. Odukoya, M. Onigbogi and O. Sekoni. Knowledge and attitude toward smoke-free legislation and second-hand smoking exposure among workers in indoor bars, beer parlors and discotheques in Osun State of Nigeria. International journal of health policy and management. 2015;4(4):229-34.
Owusu-Dabo et al.	2011	Ghana	Article	Background: Preventing an epidemic increase in smoking prevalence is a major challenge for developing countries. Ghana, has maintained a low smoking prevalence despite the presence of cigarette manufacturing for many decades. Some of this success may have been contributed by cultural factors and attitudes. We have studied public awareness of health risks, attitudes to smoke-free policy, tobacco advertising/promotion and other factors in a Ghanaian population sample. Methods: We used two-stage cluster randomized sampling to study household members aged 14 and over in a representative household sample in the Ashanti Region of Ghana. Results: 6258 people, 88% of those eligible, took part in the study. Knowledge of health risks of smoking and passive smoking was high; radio was the main source of such information. Most people work and/or spend time in places where smoking is permitted. There was very strong support (97%) for comprehensive smoke-free legislation, particularly among Christians and Muslims. Despite the advertising ban, a third of respondents (35%), particularly in urban areas, had noticed advertising of tobacco or tobacco products, on the radio (72%) and television (28%). Among smokers, 76% had attempted to quit in the last 6 months, with the main sources of advice being friends and spouses. Use of nicotine replacement therapy was very rare. Low levels of health awareness were seen in females compared with males (Adjusted Odds Ratio (AOR); 0.51, 95% CI 0.39-0.69, $p<0.001$). High levels of health awareness was seen among Traditionalists compared with Christians (AOR; 2.16 95% CI 0.79-5.94, $p<0.05$) and the relatively well educated (AOR; 1.70 95% CI 1.12-2.58, $p<0.05$) and those living in rural areas (AOR 1.46 95% CI 1.14-1.87, $p=0.004$). Conclusion: Awareness of health risks and support for smoke-free policy are high in Ghana. Exposure to tobacco advertising or promotion is limited and most smokers have tried to quit. Whether these findings are cause or effect of current low smoking prevalence is uncertain.	E. Owusu-Dabo;S. Lewis;A. McNeill;A. Gilmore;J. Britton. Support for smoke-free policy, and awareness of tobacco health effects and use of smoking cessation therapy in a developing country. BMC Public Health. 2011;11
PAHO	2013	Multiple	Report	No abstract available.	PAHO. Manual for developing tobacco control legislation in the region of the Americas. Washington, DC: Pan American Health Organization;2013.
Pederson et al.	1991	Multiple	Article	More and more legislation regulating smoking in public places is being enacted. A conceptual model is proposed incorporating a large number of factors that may affect smokers' compliance with resulting restrictions. The model stems from findings in our own research and from a consideration of the literature. It includes individual variables such as personality characteristics and attitudes, as well as population variables such as social norms and political and economic factors. Education about the health effects of environmental tobacco smoke and attitudes toward legislative measures regulating smoking, in the context of a supportive environment, are postulated to directly affect personal compliance with regulations. Some directions for future research and implications for public policy are presented.	L. L. Pederson;J. M. Wanklin;S. B. Bull;M. J. Ashley. A conceptual framework for the roles of legislation and education in reducing exposure to environmental tobacco smoke. American J of Health Promotion. 1991;6(2):105-111.

Pederson et al.	1991	Multiple	Article	More and more legislation regulating smoking in public places is being enacted. A conceptual model is proposed incorporating a large number of factors that may affect smokers' compliance with resulting restrictions. The model stems from findings in our own research and from a consideration of the literature. It includes individual variables such as personality characteristics and attitudes, as well as population variables such as social norms and political and economic factors. Education about the health effects of environmental tobacco smoke and attitudes toward legislative measures regulating smoking, in the context of a supportive environment, are postulated to directly affect personal compliance with regulations. Some directions for future research and implications for public policy are presented.	Pederson LL, Wanklin JM, Bull SB, Ashley MJ. A conceptual framework for the roles of legislation and education in reducing exposure to environmental tobacco smoke. American journal of health promotion : AJHP. 1991;6(2):105-111.
Perkins and E et al.	2014	Multiple	Article	Objectives: We examine the determinants governing both countries' enactment of smoking bans in public places and their ability to successfully put these bans into effect. Methods: Using a large sample (N = 99-184) of low-, middle- and high-income countries, econometric techniques are used to estimate the influence of several variables on cross-national variations in the adoption and compliance of second-hand smoke laws (2010). Results: We find similarities in the determinants of adoption and compliance. Yet more notable are the differences, with several political economy factors which have a statistically significant influence on countries' level of compliance with existing smoke-free laws in public places found not to consistently influence their propensity to adopt bans in the first place. Possible explanations for this discrepancy are that governments are motivated to adopt smoking bans for reasons other than protecting the health of their citizens and that the real costs of smoking bans are predominantly borne at the compliance stage. Conclusions: More effort needs to be made to ensure that governments realize their existing policy commitments through effective enforcement of bans.	R. Perkins and E. Neumayer. Adoption and compliance in second-hand smoking bans: a global econometric analysis. International journal of public health. 2014;59(5):859-66.
Persai et al.	2016	India	Article	Introduction. While extensive scientific evidence exists on the tobacco epidemic, a lack of understanding of both policies and their appropriate way of implementation continues to hinder effective tobacco control. This is especially so in the developing countries such as India. The present study aims to understand current implementation practices and the challenges faced in mainstreaming tobacco control policy and program. Methods. We chose a qualitative study design to conduct the case analysis. A total of 42 in-depth interviews were undertaken with seven district officials in six districts of Andhra Pradesh. A conceptual framework was developed by applying grounded theory for analysis. Analysis was undertaken using case analysis approach. Results and Discussion. Our study revealed that most program managers were unfamiliar with the comprehensive tobacco control policy. Respondents have an ambiguous opinion regarding integration of tobacco control program into existing health and development programs. Respondents perceive lack of resources, low prioritization of tobacco control, and lack of monitoring and evaluation of smoke-free laws as limiting factors affecting implementation of tobacco control policy. Conclusion. The findings of this study highlighted the need for a systematic, organized action plan for effective implementation of tobacco control policy and program.	D. Persai, R. Panda and A. Gupta. Examining Implementation of Tobacco Control Policy at the District Level: A Case Study Analysis from a High Burden State in India. Advances in preventive medicine. 2016;2016():4018023.
Radwan et al.	2012	Egypt	Article	SETTING: A smoke-free law was passed in Egypt in 2007. In 2010 a bylaw was issued, leading to a drive by the Ministry of Health and Population (MOHP) to launch a smoke-free initiative in Alexandria, the second largest city. Objective: To assess public opinion with regard to 100% smoke-free legislation and its implementation in the Alexandria governorate. DESIGN: The Union Middle-East Office, in collaboration with the Central Agency for Public Mobilization and Statistics and the MOHP, conducted a cross-sectional survey among 427 randomly selected adults (206 males and 221 females), covering the seven major districts of the Alexandria governorate. Results: The majority of the interviewed subjects (98%) expressed support of the government in enacting 100% smoke-free indoor legislation in all public places and public transport. Respondents endorsed the government plan to implement legislation imposing 100% smoke-free public places. More than one third (33.5%) of all respondents indicated that they would increase visits to restaurants if they were smoke-free, and 63% indicated no impact at all. Conclusion: The results of the poll clearly support results from different countries worldwide that smoke-free policies are popular and supported by the public. (copyright) 2012 The Union.	G. N. Radwan;A. H. Emam;K. M. Maher;M. Mehrez;N. El-Sayed;G. M. El-Nahas. Public opinion on smoke-free policies among Egyptians. Int J of Tuberculosis and Lung Disease. 2012;16(10):1412-1417.
Rashid et al.	2014	Malaysia	Article	This cross sectional survey was conducted to determine the support in making Penang UNESCO World Heritage Site (GTWHS) smoke free and to determine the influence of tolerance towards smoking on this support. This is the first phase in making Penang, Malaysia a smoke free state. A multistage sampling process was done to select a sample of respondents to represent the population of GTWHS. Attitude towards smoking was assessed using tolerance as a proxy. A total of 3,268 members of the community participated in the survey. A big majority (n = 2969; 90.9%) of the respondents supported the initiative. Support was lowest among the owners and residents/tenants, higher age groups, the Chinese, men, respondents who had poor knowledge of the places gazetted as smoke free, and respondents with poor knowledge of the health effects on smokers and on passive smokers. The odds (both adjusted and unadjusted) of not supporting the initiative was high among those tolerant to smoking in public areas. Tolerance towards smoking was associated with 80.3% risk of non-support in the respondents who were tolerant to smoking and a 57.2% risk in the population. Health promotion and education concerning the harm of tobacco smoke in Malaysia, which has mainly targeted smokers, must change. Health education concerning the risks of second hand smoke must also be given to non-smokers and efforts should be made to denormalize smoking.	A. Rashid, A. Ab Manan, N. Yahya and L. Ibrahim. The Support for Smoke Free Policy and How It Is Influenced by Tolerance to Smoking - Experience of a Developing Country. . 2014;9(10):.
Ross et al.	2005	Multiple	Report	No abstract available.	Ross H, Joossens L. Smoke free Europe makes economic sense. Smoke Free Europe partnership;-May 2005.

Sadykova et al.	2011	Kazakh-stan	Report	No abstract available.	Sadykova J. WHO smoke-free city case study: Almaty - the first smoke-free city in the post Soviet region. Geneva: World Health Organization;2011.
Samet et al.	1998	Multiple	Article	No abstract available.	Samet JM, Yach D, Taylor C, Becker K. Research for effective global tobacco control in the 21st century: report of a working group convened during the 10th World Conference on Tobacco or Health. Tobacco control. 1998;7(1):72-77.
Schick et al.	2008	Georgia	Article	No abstract available.	S. Schick, K. Gvinianidze, D. Tser-eteli, T. Novontny, K. Hammong. Pilot study of compliance with healtcare facility smoking laws in Georgia. Georgian Med News. 2008;(206):47-50.
Schoj et al.	2012	Argen-tina	Article	Background There is scarce evidence of secondhand smoke (SHS) and disparity in developing countries.We evaluated the relationship between so-cio-demographic variables and secondhand smoke-related factors in Argentina. Methods We conducted a randomized telephone survey (2008/2009) in 25 Argentinean cities. We included a sample of 160 respondents per city stratified by sex and age. We used different generalized multivariate regression models with a confidence interval of 95 % for the five outcome variables. Results We sampled 4,000 respondents, 52.2 % women, 36 % adolescents and young adults (15-29 years), 58 % C12 years of education, and 72.6 % nonsmokers. Support to 100 % smoke-free environment legislation was higher in older than in younger respondents, OR = 1.5 (IC: 1.2-2.0), and in people with higher education levels, OR = 1.2 (IC: 1.1-1.4). Exposure to SHS was sig-nificantly lower in men than in women at home and in public places, IRR = 0.7 (IC: 0.5-0.9) and IRR = 0.8 (IC: 0.6-0.9), respectively. Older respondents reported lower exposure at home and in public places than adolescents and young adults, IRR = 0.6 (IC: 0.4-0.8) and IRR = 0.4 (IC: 0.3-0.5), respectively. People with higher education levels had a higher level of exposure in indoor public places than less educated people, IRR = 1.1 (IC: 1.1-1.2). Knowledge of respiratory disease in children caused by SHS exposure was lower in men than in women, RRR = 0.3 (IC: 0.1-0.6). Perceived compliance was higher in men than in women, OR = 1.4 (IC: 1.1-1.8) and in people with higher education levels, OR = 1.2 (IC: 1.1-1.4). Older and more educated respondents were more empowered than. younger and less educated people, OR = 1.5 (IC: 1.2-1.9) and OR = 1.2 (IC: 1.1-1.3), respectively. Reference groups for each variable were age: 15-29; education: B7 years; and sex: men. Conclusions This is the first study to explore sociodemographic variables regarding secondhand smoke in our country. Women and younger people are more vulnerable to SHS-related factors in Argentina. (copyright) 2012 Springer Science+Business Media B.V.	V. Schoj;L. Allemandi;O. Ianovsky;M. Lago;M. Alderete. Smoke-free environments: Age, sex, and educational dispar-ity in 25 Argentinean cities. Cancer Causes and Control. 2012;23(10):1607-1614.
Schoj et al.	2010	Argen-tina	Article	Objective: To evaluate indoor air pollution in hospitality venues in Argentina. Material and Methods: PM2.5 levels were measured in a convenience sample of venues in 15 cities with different legislative contexts following a protocol developed by Roswell Park Cancer Institute. Results: 554 samples were collected. Across all 5 smokefree cities the mean PM2.5 level was lower during daytime vs. evening hours, 24 vs. 98 PM2.5 respectively (p=.012). In the three cities evaluated before and after legislation, PM2.5 levels decreased dramatically (p<0.001 each). Overall, PM2.5 levels were 5 times higher in cities with no legislation vs. smokefree cities (p<0.001). In cities with designated smoking areas, PM2.5 levels were not statistically different between smoking and non-smoking areas (p=0.272). Non-smoking areas had significantly higher PM2.5 levels compared to 100% smokefree venues in the same city (twofold higher) (p=0.017). Conclusions: Most of the participating cities in this study had significantly lower PM2.5 levels after the implementation of 100% smokefree legislation. Hence, it represents a useful tool to promote 100% smokefree policies in Argentina.	V. Schoj;E. M. Sebrié;M. E. Pizarro;A. Hyland;M. J. Travers. Informing effective smoke-free policies in Argentina: Air quality monitoring study in 15 cities (2007-2009). Salud Publica Mex. 2010;52(SUPPL.2):S157-S167.
Sebrie et al.	2012	Reg-Lat-in Amer-ica	Article	We reviewed the adoption and implementation of smokefree policies in all Latin American and the Caribbean (LAC) countries. Significant progress has been achieved among LAC countries since the WHO Framework Convention on Tobacco Control (FCTC) was adopted in 2005. Both national and sub-national legislation have provided effective mechanisms to increase the fraction of the population protected from secondhand tobacco smoke. Civil society has actively promoted these policies and played a main role in enacting them and monitoring their enforcement. The tobacco industry, while continuing to oppose the approval and regulation of the laws at legislative and executive levels, has gone a step further by litigating against them in the Courts. As in the US and elsewhere, this litigation has failed to stop the legislation. (copyright) 2012 by the authors; licensee MDPI, Basel, Switzerland.	E. M. Sebrie;V. Schoj;M. J. Travers;B. McGaw;S. A. Glantz. Smokefree policies in Latin America and the Caribbe-an: Making progress. Int J Env Res and Public Health. 2012;9(5):1954-1970.

Sebrie et al.	2010	Argen-tina	Article	Objective: To describe the process of approval and implementation of a comprehensive smoke-free law in the province of Santa Fe, Argentina, between 2005 and 2009. Methods: Review of the Santa Fe smoke-free legislation, articles published in local newspapers and documentation on two lawsuits filed against the law, and interviews with key individuals in Santa Fe. Results: Efforts to implement smoke-free policies in Santa Fe began during the 1990s without success, and resumed in 2005 when the provincial Legislature approved the first 100% smoke-free subnational law in Argentina. There was no strong opposition during the discussions within the legislature. As in other parts of the world, pro-tobacco industry interests attempted to block the implementation of the law using well known strategies. These efforts included a controversy media campaign set up, the creation of a hospitality industry association and a virtual smokers' rights group, the introduction of a counterproposal seeking modification of the law, the challenge of the law in the Supreme Court, and the proposal of a weak national bill that would 'conflict' with the subnational law. Tobacco control advocates sought media attention as a strategy to protect the law. Conclusions: Santa Fe is the first subnational jurisdiction in Latin America to have enacted a comprehensive smoke-free policy following the recommendations of the World Health Organization (WHO) Framework Convention on Tobacco Control. After 3 years of implementation, pro-tobacco industry forces failed to undermine the law. Other subnational jurisdictions in Argentina, as well as in Mexico and Brazil are following the Santa Fe example.	E. M. Sebrie;S. A. Glantz. Local smoke-free policy development in Santa Fe, Argentina. Tob Control. 2010;19(2):110-6.
Sharifi et al.	2012	Iran	Article	Background: Iran signed the Framework Convention on Tobacco Control on June 16, 2003 and it was ratified by the parliament and the House of Representatives on November 6, 2005. Finally, it came into force on February 4, 2006. In this study, we aimed to evaluate health experts' opinion about tobacco control activities in Iran. Materials and Methods: This was a qualitative case study. We used a series of openended questionnaires to assess important information regarding Iranian National Tobacco Control law and FCTC implementation. The study population comprised of health experts. Use of this method ensured the validity of questionnaires' contents. The first round of the questionnaire had been pre-tested in a pilot study. The final structure and lay out of questionnaires consisted of three main parts. The first part was designed with 7 multiple choice questions. Participants were able to rank answers from five (the most important) to one (the least important). The second part comprised four questions mainly on National Tobacco Control Program (NTCP) and the final part was about FCTC. Data collection was carried out between May 2010 and May 2011. In the analysis process each interview was considered as a separate case and then compared to other cases to ascertain variations in answers. Results: All 40 members (100%) of the panel completed the entire process. All the participants had a consensus on tobacco control program in Iran. They believed the prevention programs to be important priorities in this regard. Tobacco Company as a governmental organization is believed to be the main barrier against tobacco control activities in Iran, and banning sales of tobacco to minors and controlling its smuggling are important factors for decreasing the supply of tobacco products. It is essential to implement comprehensive tobacco control law in Iran. Conclusion: It is essential to implement comprehensive tobacco control law in Iran that covers all the priorities mentioned above. Considering the chronological aspect of law implementation, priority should be given to the more important parts of the law. (copyright) 2012 NRITLD.	H. Sharifi;Z. Hessami;M. R. Masjedi. Health experts' opinions about tobacco control activities in iran: Results from a delphi panel of national experts. Tanaffos. 2012;11(4):50-55.
Thrasher et al.	2013	Multiple	Article	Objective: To determine the prevalence, correlates and changes in secondhand smoke (SHS) exposure over the period after comprehensive smoke-free policy implementation in two Latin American countries. Methods: Data were analysed from population-based representative samples of adult smokers and recent quitters from the 2008 and 2010 waves of the International Tobacco Control Policy Evaluation Survey in Mexico (n = 1766 and 1840, respectively) and Uruguay (n = 1379 and 1411, respectively). Prevalence of SHS exposure was estimated for regulated venues, and generalized estimating equations were used to determine correlates of SHS exposure. Results: Workplace SHS exposure in the last month was similar within and across countries (range: Mexico 20-25%; Uruguay 14-29%). At the most recent restaurant visit, SHS exposure was lower where comprehensive smoke-free policies were implemented (range: Uruguay 6-9%; Mexico City 5-7%) compared with Mexican cities with weaker policies, where exposure remained higher but decreased over time (32-17%). At the most recent bar visit, SHS exposure was common (range: Uruguay 8-36%; Mexico City 23-31%), although highest in jurisdictions with weaker policies (range in other Mexican cities: 74-86%). In Uruguay, males were more likely than females to be exposed to SHS across venues, as were younger compared with older smokers in Mexico. Conclusions: Comprehensive smoke-free policies are more effective than weaker policies, although compliance in Mexico and Uruguay is not as high as desired.	J. F. Thrasher;E. Nayeli Abad-Vivero;E. M. Sebrie;T. Barrientos-Gutierrez;M. Boado;H. H. Yong;E. Arillo-Santillan;E. Bianco. Tobacco smoke exposure in public places and workplaces after smoke-free policy implementation: a longitudinal analysis of smoker cohorts in Mexico and Uruguay. Health Policy Plan. 2013;28(8):789-98.

Thrasher et al.	2011	Multiple	Article	Reports an error in "Smoke-free policies and the social acceptability of smoking in Uruguay and Mexico: Findings from the International Tobacco Control Policy Evaluation Project" by James F. Thrasher, Marcelo Boado, Ernesto M. Sebrié and Eduardo Bianco (Nicotine & Tobacco Research, 2009[Jun], Vol 11[6], 591-599). The article "Smoke-free policies and the social acceptability of smoking in Uruguay and Mexico: Findings from the International Tobacco Control Policy Evaluation Project", involved analysis of data from Uruguay. Follow-up data have since been collected, and it was determined that one Uruguayan interviewer had "faked" results. After eliminating all data collected by this interviewer, approximately 10% of the Uruguayan sample was lost, going from 1,002 to 887 participants. We have rerun the analyses from the published paper, and the vast majority of the results are the same. A few of the marginally significant or nonsignificant results have changed; however, these changes do not affect the primary conclusions of the paper. (The following abstract of the original article appeared in record 2010-19443-002). Introduction: Little research has been conducted to determine the psychosocial and behavioral impacts of smoke-free policies in middle-income countries. Methods: Cross-sectional data were analyzed from the 2006 waves of the International Tobacco Control Policy Evaluation. Survey comparing adult smokers in Mexico (n = 1,080), where smoke-free legislation at that time was weak, and Uruguay (n = 1,002), where comprehensive smoke-free legislation was implemented. Analyses aimed to determine whether exposure to smoke-free policies and perceived antismoking social norms were associated with smokers ' receiving cues about the bothersome nature of secondhand smoke (SHS), with smokers ' reactance against such cues, and with smokers ' level of support for smoke-free policies in different venues. Results: In bivariate analyses, Uruguayan smokers were more likely than Mexican smokers to experience verbal anti-SHS cues, lower reactance against anti-SHS cues, stronger antismoking societal norms, and stronger support for 100% smoke-free policies in enclosed workplaces, restaurants, and bars. In multivariate models for both countries, the strength of voluntary smoke-free policies at home was independently associated with support for smoke-free policies across all venues queried, except for in bars among Uruguayans. Perceived strength of familial antismoking norms was consistently associated with all indicators of the social acceptability of smoking in Uruguay but only with the frequency of receiving anti-SHS verbal cues in Mexico. Discussion: These results are generally consistent with previous research indicating that comprehensive smoke-free policies are likely to increase the social unacceptability of smoking and that resistance against such policies is likely to diminish once such policies are in place. (PsycINFO Database Record (c) 2012 APA, all rights reserved)	James F. Thrasher;Marcelo Boado;Ernesto M. Sebrié;Eduardo Bianco. Smoke-free policies and the social ERROR acceptability of smoking in Uruguay and Mexico: Findings from the International Tobacco Control Policy Evaluation Project': Erratum. Nicotine & Tobacco Research. 2011;13(4):298-300.
Thrasher et al.	2010	Mexico	Article	Objectives: We assessed attitudes and beliefs about smoke-free laws, compliance, and secondhand smoke exposure before and after implementation of a comprehensive smoke-free law in Mexico City. Methods: Trends and odds of change in attitudes and beliefs were analyzed across 3 representative surveys of Mexico City inhabitants: before implementation of the policy (n=800), 4 months after implementation (n=961), and 8 months after implementation (n=761). Results: Results indicated high and increasing support for 100% smoke-free policies, although support did not increase for smoke-free bars. Agreement that such policies improved health and reinforced rights was high before policy implementation and increased thereafter. Social unacceptability of smoking increased substantially, although 25% of nonsmokers and 50% of smokers agreed with smokers' rights to smoke in public places at the final survey wave. Secondhand smoke exposure declined generally as well as in venues covered by the law, although compliance was incomplete, especially in bars. Conclusions: Comprehensive smoke-free legislation in Mexico City has been relatively successful, with changes in perceptions and behavior consistent with those revealed by studies conducted in high-income countries. Normative changes may prime populations for additional tobacco control interventions.	J. F. Thrasher;R. Perez-Hernandez;K. Swayampakala;E. Arillo-Santillan;M. Bottai. Policy support, norms, and secondhand smoke exposure before and after implementation of a comprehensive smoke-free law in Mexico city. Am J Public Health. 2010;100(9):1789-98.
Thrasher et al.	2010	Mexico	Article	The World Health Organization's Framework Convention on Tobacco Control promotes comprehensive smoke-free laws. The effective implementation of these laws requires citizen participation and support. Risk communication research suggests that citizens' perceptions of the fairness of smoke-free laws would help explain their support for the law. This study aimed to assess the factors that correlate with citizens' perceptions of the distributive, procedural and interpersonal justice of smoke-free laws, as well as how these perceptions are related to support for and intention to help enforce these laws. Study data came from a cross-sectional, population-based survey of 800 Mexico City inhabitants before a comprehensive smoke-free policy was implemented there in 2008. Structural equation modeling was used to estimate the bivariate and multivariate adjusted paths relating study variables. In the final multivariate model, the three justice concepts mediated the influence of smoking status, perceived dangers of secondhand smoke exposure, strength of home smoking ban, and perceived rights of smokers on the two distal constructs of support for smoke-free policy and intention to help enforce it. Statistically significant paths were estimated from distributive and procedural justice to support for the law and intention help enforce it. The path from interpersonal justice to support for the law was not significant, but the path to intention to help enforce the law was. Finally, the path from support for the law to the intention to enforce it was statistically significant. These results suggest that three distinct dimensions of perceived justice help explain citizen support for smoke-free policies. These dimensions of perceived justice may explain the conditions under which smoke-free policies are effectively implemented and could help shape the focus for communication strategies that aim to ensure effective implementation of this and other public health policies. (copyright) 2009 Elsevier Ltd. All rights reserved.	J. F. Thrasher;J. C. Besley;W. Gonzalez. Perceived justice and popular support for public health laws: A case study around comprehensive smoke-free legislation in Mexico City. SSM. 2010;70(5):787-793.

Thrasher et al.	2009	Multiple	Article	Introduction: Little research has been conducted to determine the psychosocial and behavioral impacts of smoke-free policies in middle-income countries. Methods: Cross-sectional data were analyzed from the 2006 waves of the International Tobacco Control Policy Evaluation. Survey comparing adult smokers in Mexico (n = 1,080), where smoke-free legislation at that time was weak, and Uruguay (n = 1,002), where comprehensive smoke-free legislation was implemented. Analyses aimed to determine whether exposure to smoke-free policies and perceived antismoking social norms were associated with smokers' receiving cues about the bothersome nature of secondhand smoke (SHS), with smokers' reactance against such cues, and with smokers' level of support for smoke-free policies in different venues. Results: In bivariate analyses, Uruguayan smokers were more likely than Mexican smokers to experience verbal anti-SHS cues, lower reactance against anti-SHS cues, stronger antismoking societal norms, and stronger support for 100% smoke-free policies in enclosed workplaces, restaurants, and bars. In multivariate models for both countries, the strength of voluntary smoke-free policies at home was independently associated with support for smoke-free policies across all venues queried, except for in bars among Uruguayans. Perceived strength of familial antismoking norms was consistently associated with all indicators of the social acceptability of smoking in Uruguay but only with the frequency of receiving anti-SHS verbal cues in Mexico. Discussion: These results are generally consistent with previous research indicating that comprehensive smoke-free policies are likely to increase the social unacceptability of smoking and that resistance against such policies is likely to diminish once such policies are in place. (copyright) The Author 2009. Published by Oxford University Press on behalf of the Society for Research on Nicotine and Tobacco. All rights reserved.	J. F. Thrasher;M. Boado;E. M. Sebrie;E. Bianco. Smoke-free policies and the social acceptability of smoking in Uruguay and Mexico: Findings from the International Tobacco Control Policy Evaluation Project. Nicotine & Tobacco Research. 2009;11(6):591-599.
Thrasher et al.	2008	Mexico	Article	The World Health Organization Framework Convention on Tobacco Control (WHO-FCTC) promotes the implementation of best-practices tobacco control policies at a global scale. This article describes features of the sociocultural and political-economic context of Mexico that pose challenges and opportunities to the effective translation of WHO-FCTC policies there. It also considers how strategic communication efforts may advance these policies by framing their arguments in ways that resonate with prevalent values, understandings, and concerns. A focus on a smoke-free policy illustrates barriers to policy compliance, including how similar issues have been overcome among Latino populations in California. Overall, this article aims to lay the foundation for comparative research from policy uptake to impact so that the scientific evidence base on tobacco control policies includes examination of how context moderates this process.	J. F. Thrasher;L. M. Reynales-Shigematsu;L. Baezconde-Garbanati;V. Villalobos;P. Tellez-Giron;E. Arillo-Santillan;A. Dorantes-Alonso;R. Valdes-Salgado;E. Lazcano-Ponce. Promoting the effective translation of the framework convention on tobacco control: a case study of challenges and opportunities for strategic communications in Mexico. Eval Health Prof. 2008;31(2):145-66.
Thrasher et al.	2006	Multiple	Article	No abstract available.	Thrasher JF, Chaloupka F, Hammond D, et al. [Evaluating tobacco control policy in Latin American countries during the era of the Framework Convention on Tobacco Control]. Salud Publica Mex. 2006;48 Suppl 1:S155-166.
Travers et al.	2015	India	Article	Tobacco smoke has compounds that are known as human carcinogens. With every breath of secondhand smoke we inhale thousands of chemicals. The Government of India in the interest of public health has enacted the Cigarette and Other Tobacco Products Act (COTPA), 2003, which bans smoking in all the public places including hotels and restaurants. The purpose of this study was to observe and record air pollution in smoke free and smoke observed locations and thereby find out whether the owners/managers of hotels, restaurants, and bars comply with rules of COTPA. Objectives: The objectives of the study were to measure and compare the level of particulate air pollution from secondhand smoke (PM2.5) in smoking and nonsmoking venues. MATERIALS AND Methods: The study was conducted from September 2009 to March 2010 in Karnataka, India following a nonrandom sample of 79 locations, which included restaurants, bars, cafes, hotels, and tea stalls in two districts. The concentration of PM2.5was measured using a TSI SidePak AM510 Personal Aerosol Monitor. Results: In Karnataka out of the 79 hospitality locations, smoking was observed in 58% places and only 28% had displayed the required 'No Smoking' signage. Places where indoor smoking was observed had high levels of air pollution with average 135 PM2.5, which were 3.1 times higher than the average 43 PM2.5in smoke-free locations and 14 times higher than the World Health Organization (WHO) target air quality guideline for PM2.5. The average PM2.5levels in different locations ranged from 11 to 417 μg/m3 and was lower in the case of apparently compliant designated smoking area (DSR). Conclusions: The patrons and the workers in the hospitality sector continue to be exposed to secondhand smoke despite the enactment of COTPA, which bans smoking in public places. This situation demands stringent measures for effective implementation of the Smoke Free Act and negative response to smoking among civil society.	M. Travers, N. Nayak, V. Annigeri and N. Billava. Indoor air quality due to secondhand smoke: Signals from selected hospitality locations in rural and urban areas of Bangalore and Dharwad districts in Karnataka, India. . 2015;52(4):708-713.

Turan et al.	2014	Turkey	Article	Introduction: The aim of the study is to determine the views about law, change of second hand smoke (SHS) quantity, people's smoking related behaviours after the publishment of law which prohibits use of tobacco products in closed public areas. Materials and Methods: Questionnaire including 47 questions was performed to 600 people, who were selected by Turkey Statistics Institution to present population of Izmir. Results: 53.7% of participants were female and 46.3% was male; the mean age was 41.5±15.6. 98% of all participants were aware of the law and 91.5% of them were supporting it. 42% of participants who succeeded in quitting had managed it after the ban. Restriction of areas, rise of prices and effects of SHS were the most popular reasons of reducing/quitting smoking after the law. Nonsmokers were exposed to SHS at homes; 35.1%, at vehicles: 14.9%, at work places: 14.4%. The number of smoking cigarettes was reduced by 3.19 cigarettes/day. The rate of violation of the ban was 32.3%; only 21.3% of violators were warned. 96.4% of law supporters found law successful and sufficient. Conclusion: A big amount of participants thinks about quitting in Izmir; law is thought to have important effect on this decision. SHS has reduced mostly in public areas with ban, but law must be enlarged to prevent SHS at private areas such as houses, vehicles and workplaces. As a result; the ban encouraged smokers to quit and reduced SHS at public areas. We believe that paying more attention for law sanction and extending the borders of law and reviewing the ban, will make our country more smoke free from now.	P. A. Turan, G. Ergör, O. Turan, S. Doganay and O. Klnc. The changes in smoking related behaviours and second hand smoke after the smoking ban in Izmir. 2014;62(1):27-38.
Turner et al.	2016	India	Article	SETTING: India's Cigarettes and Other Tobacco Products Act (COTPA) prohibits smoking in public places, limits advertising at points of sale, and bans sales in close proximity to educational institutions. Objective : To examine key stakeholders' (i.e., tobacco retailers, hotel and restaurant owners/managers, law enforcement officials, school principals/administrators) perceptions of facilitators and barriers to compliance with COTPA. DESIGN: Using semi-structured in-depth interviews (n= 60), we identified a range of facilitators and barriers that varied across the venues. Results : A number of simple measures to increase compliance were identified: provide signs to retailers, provide aesthetically pleasing signs more fitting for the atmosphere of the hotel/restaurant, etc., and empower law enforcement to implement the law. Conclusions : Priority should be placed on public awareness, as it can facilitate compliance in numerous venues. Communication efforts could be undertaken to change the public's residual negative attitudes toward the policy.	M. M. Turner, R. N. Rimal, E. Lumby, J. Cohen, A. Surette, V. Roundy, E. Feighery and V. Shah. Compliance with tobacco scontrol policies in India: An examination of facilitators and barriers. . 2016;20(3):411-416.
USDHHS et al.	2006	Multiple	Report	No abstract available.	USDHHS. The health consequences of involuntary exposure to tobacco smoke: A report of the Surgeon General. Atlanta: USDHHS, CDC, Coordinating Center on Health Promotion, National Center for Chronic Disease and Health Promotion, Office on Smoking and Health;2006.
Veeranki et al.	2015	Multiple	Article	PURPOSE: To estimate the prevalence of secondhand smoke (SHS) exposure among never-smoking adolescents and identify key factors associated with such exposure. Methods: Data were obtained from nationally representative Global Youth Tobacco Surveys conducted in 168 countries during 1999-2008. SHS exposure was ascertained in relation to the location-exposure inside home, outside home, and both inside and outside home, respectively. Independent variables included parental and/or peer smoking, knowledge about smoke harm, attitudes toward smoking ban, age, sex, and World Health Organization region. Simple and multiple logistic regression analyses were conducted. Results: Of 356,414 never-smoking adolescents included in the study, 30.4%, 44.2%, and 23.2% were exposed to SHS inside home, outside home, and both, respectively. Parental smoking, peer smoking, knowledge about smoke harm, and positive attitudes toward smoke ban were significantly associated with increased odds of SHS exposure. Approximately 14% of adolescents had both smoking parents and peers. Compared with never-smoking adolescents who did not have both smoking parents and peers, those who had both smoking parents and peers had 19 (adjusted odds ratio [aOR], 19.0; 95% confidence interval [CI], 16.86-21.41), eight (aOR, 7.71; 95% CI, 7.05-8.43), and 23 times (aOR, 23.16; 95% CI, 20.74-25.87) higher odds of exposure to SHS inside, outside, and both inside and outcome home, respectively. Conclusions: Approximately one third and two fifths of never-smoking adolescents were exposed to SHS inside or outside home, and smoking parents and/or peers are the key factors. Study findings highlight the need to develop and implement comprehensive smoke-free policies consistent with the World Health Organization Framework Convention on Tobacco Control.	S. P. Veeranki, H. M. Mamudu, S. Zheng, R. M. John, Y. Cao, D. Kioko, J. Anderson and A. E. Ouma. Secondhand smoke exposure among never-smoking youth in 168 countries. The Journal of adolescent health : official publication of the Society for Adolescent Medicine. 2015;56(2):167-73.
Villarreiz et al.	2011	India	Report	No abstract available.	Villarreiz D. WHO smoke-free city case study: Advancing the enforcement of the smoking ban in public places – Davao City, Philippines. Geneva: World Health Organization;2011.
WHO	2009	Multiple	Report	No abstract available.	World Health Organization. WHO Framework Convention on Tobacco Control; Geneva, Switzerland: WHO Press, 2003.

WHO	1999	Multiple	Report	No abstract available.	WHO Tobacco Free Initiative & Research for International Tobacco Control. (1999) . Confronting the epidemic : a global agenda for tobacco control research, July 1999, Geneva, Switzerland. World Health Organization. http://www.who.int/iris/handle/10665/66028
WHO	2011	Multiple	Article	This publication is the result of a joint project between the WHO Tobacco Free Initiative (TFI) and the WHO Centre for Health Development (Kobe Centre). It is intended as a technical resource to assist Member States implementing Article 8 of the WHO Framework Convention on Tobacco Control (WHO FCTC) and its guidelines in order to fully protect people from tobacco smoke in cities. Guidelines on choosing action priorities and where and how long to implement smoke-free legislation are provided. The key elements for successful implementation of smoke-free legislation and the 12 steps to a smoke-free city are discussed. This document is addressed to mayors and their staff, and other city officials to prepare and implement smoke-free legislation that is popular, complied with, and effective in improving health.	. Making cities smoke-free. report. 2011;(20 Avenue Appia, 1211 Geneva 27, Switzerland.):viii-pp.
WHO	2007	Multiple	Article	This publication examines lessons learned in policy-making and implementation of programmes aimed at protection from exposure to second-hand tobacco smoke. It also presents recommendations aimed at protecting workers and the public from exposure to second-hand tobacco smoke: removal of the pollutant; enacting legislation; enforcing laws; and implementation of education strategies.	. Protection from exposure to second-hand tobacco smoke: policy recommendations. report. 2007;(Avenue Appia 20, 1211 Geneva 27, Switzerland.):.
WHO	2007	Multiple	Report	No abstract available.	World Health Organization. WHO Framework Convention on Tobacco Control: Guidelines for implementation Article 8, Appendix. Link to Example Legislation. Geneva, Switzerland: WHO Press, 2009.
WHO	2007	Multiple	Report	No abstract available.	World Health Organization. WHO Framework Convention on Tobacco Control: Guidelines for implementation Article 8; Geneva, Switzerland: WHO Press, 2009.
WHO	2011	Multiple	Report	No abstract available.	World Health Organization & International Union against Tuberculosis and Lung Disease. (2011) . Building capacity for tobacco control : training package. Partcipant's Workbook. World Health Organization. http://www.who.int/iris/handle/10665/44565
WHO	2011	Multiple	Report	No abstract available.	World Health Organization & International Union against Tuberculosis and Lung Disease. (2011) . Building capacity for tobacco control : training package.Facilitator's Guide. World Health Organization. http://www.who.int/iris/handle/10665/44565

WHO	2011	Multiple	Report	No abstract available.	World Health Organization & International Union against Tuberculosis and Lung Disease. (‛ 2011)‛ . Building capacity for tobacco control : training package. Technical Guide. World Health Organization. http://www.who.int/iris/handle/10665/44565
WHO	2013	Multiple	Report	No abstract available.	WHO. Making your city smoke-free: workshop package. Participant's workbook. Geneva: World Health Organization;2013.
WHO	2013	Muizople	Report	No abstract available.	WHO. Making your city smoke-free: workshop package. Workshop Gude. Geneva: World Health Organization;2013.
Wong et al.	2011	Multiple	Article	Background: Legislation is one of the most powerful weapons for improving population health and is often used by policy and decision makers. Little research exists to guide them as to whether legislation is feasible and/or will succeed. We aimed to produce a coherent and transferable evidence based framework of threats to legislative interventions to assist the decision making process and to test this through the 'case study' of legislation to ban smoking in cars carrying children. Methods: We conceptualised legislative interventions as a complex social interventions and so used the realist synthesis method to systematically review the literature for evidence. 99 articles were found through searches on five electronic databases (MEDLINE, HMIC, EMBASE, PsychINFO, Social Policy and Practice) and iterative purposive searching. Our initial searches sought any studies that contained information on smoking in vehicles carrying children. Throughout the review we continued where needed to search for additional studies of any type that would conceptually contribute to helping build and/or test our framework. Results: Our framework identified a series of transferable threats to public health legislation. When applied to smoking bans in vehicles; problem misidentification; public support; opposition; and enforcement issues were particularly prominent threats. Our framework enabled us to understand and explain the nature of each threat and to infer the most likely outcome if such legislation were to be proposed in a jurisdiction where no such ban existed. Specifically, the micro-environment of a vehicle can contain highly hazardous levels of second hand smoke. Public support for such legislation is high amongst smokers and non-smokers and their underlying motivations were very similar - wanting to practice the Millian principle of protecting children from harm. Evidence indicated that the tobacco industry was not likely to oppose legislation and arguments that such a law would be 'unenforceable' were unfounded. Conclusion: It is possible to develop a coherent and transferable evidence based framework of the ideas and assumptions behind the threats to legislative intervention that may assist policy and decision makers to analyse and judge if legislation is feasible and/or likely to succeed.	G. Wong;R. Pawson;L. Owen. Policy guidance on threats to legislative interventions in public health: a realist synthesis. BMC Public Health. 2011;11():222.
Yang et al.	2015	China	Article	The non-communicable disease burden in China is enormous, with tobacco use a leading risk factor for the major non-communicable diseases. The prevalence of tobacco use in men is one of the highest in the world, with more than 300 million smokers and 740 million non-smokers exposed to second-hand smoke. In the past decade public awareness of the health hazards of tobacco use and exposure to second-hand smoke has grown, social customs and habits have changed, aggressive tactics used by the tobacco industry have been revealed, and serious tobacco control policies have been actively promoted. In 2014, national legislators in China began actively considering national bans on smoking in public and work places and tobacco advertising. However, tobacco control in China has remained particularly difficult because of interference by the tobacco industry. Changes to the interministerial coordinating mechanism for implementation of the WHO Framework Convention on Tobacco Control are now crucial. Progress towards a tobacco-free world will be dependent on more rapid action in China.	G. Yang, Y. Wang, Y. Wu, J. Yang and X. Wan. The road to effective tobacco control in China. Lancet (London, England). 2015;385(9972):1019-28.
Yang et al.	2015	India	Article	Aim: To compare air nicotine levels in public places in Ahmedabad, India, before (June 2008) and after (January, 2010) the implementation of a comprehensive smoking ban which was introduced in October 2008. Materials and Methods: Air nicotine concentrations were measured by sampling of vapor-phase nicotine using passive monitors. In 2008 (baseline), monitors were placed for 5-7 working days in 5 hospitals, 10 restaurants, 5 schools, 5 government buildings, and 10 entertainment venues, of which 6 were hookah bars. In 2010 (follow-up), monitors were placed in 35 similar venues for the same duration. Results: Comparison of the overall median nicotine concentration at baseline (2008) (0.06 µg/m 3 Interquartile range (IQR): 0.02-0.22) to that of follow-up (2010) (0.03 µg/m 3 IQR: 0.00-0.13), reflects a significant decline (% decline = 39.7, P = 0.012) in exposure to second-hand smoke (SHS). The percent change in exposure varied by venue-type. The most significant decrease occurred in hospitals, from 0.04 µg/m [3] at baseline to concentrations under the limit of detection at follow-up (%decline = 100, P < 0.001). In entertainment venues, government offices, and restaurants, decreases in SHS exposure also appeared evident. However, in hookah bars, air nicotine levels appeared to increase (P = 0.160). Conclusion: Overall, SHS exposure was significantly reduced in public places after the smoke-free legislation came into force. However, nicotine concentrations were still detected in most of the venues indicating imperfect compliance with the comprehensive ban.	J. Yang, B. Modi, S. Tamplin, M. Aghi, P. Dave and J. Cohen. Air nicotine levels in public places in Ahmedabad, India: Before and after implementation of the smoking ban. . 2015;40(1):27-32.

Yang et al.	2016	China	Article	Efforts toward controlling secondhand smoke in public places have been made throughout China. However, in contrast to the western world, significant challenges remain for effectively implementing smoke-free regulations. This study explores individual and regional factors which influence smoking in smoke-free public places. Participants included 16 866 urban residents, who were identified through multi-stage sampling conducted in 21 Chinese cities. The reported smoking prevalence in smoke-free public places was 41.2%. Of those who smoked in smoke-free public places, 45.9% had been advised to stop smoking. Participants stated that no-smoking warnings/signs with 'please' in the statement had a better likelihood of gaining compliance and preventing smoking in public spaces. Multilevel logistic regression analysis showed that ethnicity, education, occupation, type of smoking, age of smoking initiation, smoking situation, stress, household smoking restrictions and city population were all associated with smoking in smoke-free public places. Interestingly local smoke-free regulations were not associated with smoking in public places. The findings underscore that efforts to restrict smoking in public places in China should emphasize strong enforcement, while simultaneously raising public awareness of the perils of second hand smoke.	T. Yang, S. Jiang, R. Barnett, J. L. Oliffe, D. Wu, X. Yang, L. Yu and R. R. Cottrell. Who smokes in smoke-free public places in China? Findings from a 21 city survey. Health education research. 2016;31(1):36-47.
Ye et al.	2015	China	Article	Background: According to the partial smoke-free legislation implemented on 1 September 2010 in Guangzhou, China, smoke-free did not cover all indoor areas. Some places have a full smoking ban (100 % smoke-free), other places have a partial smoking ban, and homes have no ban. This study aimed to compare the smoking behaviors before and after implementation of a smoke-free legislation. METHOD: A repeated cross-sectional survey was conducted on smoking-related behaviors with a total of 4,900 respondents before, and 5,135 respondents after the legislation was instituted. For each wave of the survey, a three-stage stratified sampling process was used to obtain a representative sample. Pearson's Chi-square test was used to determine differences of smoking prevalence and quit ratio between the two samples. Logistic regression models were used to examine the associations of a smoke-free legislation with smoking behaviors. Results: The overall daily smoking rate declined significantly from 20.8 % to 18.2 % ($p < 0.05$), especially among those aged 15-24 years. The quit ratios increased significantly (from 14.5 % to 17.9 %), but remained low among 15-44 year olds. The overall self-reported smoking behaviors in locations with a full smoking ban decreased significantly from 36.4 % to 24.3 % with the greater drops occurring in cultural venues, public transport vehicles, and government offices. Smoking in places with partial smoking bans remained high (89.6 % vs. 90.4 %), although a slight decrease was observed in some of these areas. The implementation of a smoke-free legislation did not lead to more smoking in homes (91.0 % vs 89.4 %), but smoking in homes remained high. Conclusions: These findings highlight the urgent need for a comprehensive smoke-free legislation covering all public places in Guangzhou, simultaneously educational interventions and campaigns promoting voluntary changes in home smoking need to occur.	X. Ye, S. Chen, Z. Yao, Y. Gao, Y. Xu, S. Zhou, Z. Zhu, L. Wang and Y. Yang. Smoking behaviors before and after implementation of a smoke-free legislation in Guangzhou, China. BMC public health. 2015;15():982.
Yel et al.	2013	Cambodia	Article	No abstract available.	Yel D, Bui A, Job JS, Knutsen S, Singh PN. Beliefs about tobacco, health, and addiction among adults in Cambodia: findings from a national survey. Journal of religion and health. 2013;52(3):904-914. doi: 10.1007/s10943-011-9537-x
Yildiz et al.	2010	Turkey	Article	Objective: Taxi drivers carry significant risks related to occupational health and safety. Exposure to second-hand tobacco smoke is known to be one of those health risks. As legislations for smoke-free taxis become more widespread throughout the world, this study aimed to assess knowledge and attitude of taxi drivers on the new legislation for tobacco control introduced in Turkey recently. Methodology: The study population consisted of 135 taxi drivers from 22 different taxi stations in Ankara Turkey. Data of the descriptive study was collected in October, 2008 through face-to-face interviews with a standart questionnaire. Descriptive statistics were used to summarize data, whereas chi-square was used to compare groups. Results: All the study participants were male with a mean age of 47.2(plus or minus)11.8 years. More than half of the taxi drivers (59.3%) were found to be current smokers. Although level of knowledge and attitude of taxi drivers on the new legislation were favorable in general, some difficulties and barriers were found to be present in implementation of the ban. Most of the smoking drivers were found to continue smoking in their taxis. About 80.0% of the drivers stated their concern of losing out on clients' satisfaction if they restrict smoking in their taxis. Conclusion: Taxi drivers and clients' knowledge, attitudes and behaviors are important determinants in successful implementation of legislations for smoke-free taxis.	A. N. Yildiz;O. Karadag;M. O. Gonen;F. Gurel;B. Ilhan;M. Inel;H. Islamoglu;K. Kara. Knowledge and attitude of taxi drivers on the new legislation for smoke-free taxis: An occupational health perspective. Pakistan J of Medical Sciences. 2010;26(1):111-116.

Yong et al.	2010	Multiple	Article	This study examined support for and reported compliance with smoke-free policy in air-conditioned restaurants and other similar places among adult smokers in Malaysia and Thailand. Baseline data (early 2005) from the International Tobacco Control Southeast Asia Survey (ITC-SEA), conducted face-to-face in Malaysia and Thailand (n = 4005), were used. Among those attending venues, reported total smoking bans in indoor air-conditioned places such as restaurants, coffee shops, and karaoke lounges were 40% and 57% in Malaysia and Thailand, respectively. Support for a total ban in air-conditioned venues was high and similar for both countries (82% Malaysian and 90% Thai smokers who believed there was a total ban), but self-reported compliance with bans in such venues was significantly higher in Thailand than in Malaysia (95% vs 51%, P < .001). As expected, reporting a ban in air-conditioned venues was associated with a greater support for a ban in such venues in both countries.	H. H. Yong;K. Foong;R. Borland;M. Omar;S. Hamann;B. Sirirassamee;G. T. Fong;O. Fotuhi;A. Hyland. Support for and reported compliance among smokers with smoke-free policies in air-conditioned hospitality venues in Malaysia and Thailand: findings from the International Tobacco Control Southeast Asia Survey. Asia Pac J Public Health. 2010;22(1):98-109.
York et al.	2007	Multiple	Article	The purpose of this article is to review the literature on community readiness and assess the utility of the community readiness model (CRM) for understanding and affecting smoke-free policy development and implementation. The CRM evaluates a community's capacity for successfully developing and implementing prevention or treatment interventions. The purposes of evaluating a community's readiness are to: (a) identify the stage of readiness for policy change, and (b) determine stage-specific strategies to advance a community toward policy change.	York NL, Hahn EJ. The Community Readiness Model: evaluating local smoke-free policy development. Policy Polit Nurs Pract. 2007;8(3):184-200. doi: 10.1177/1527154407308409
Young et al.	2010	Multiple	Article	Complex, transnational issues like the tobacco epidemic are major challenges that defy analysis and management by conventional methods, as are other public health issues, such as those associated with global food distribution and climate change. We examined the evolution of indoor smoke-free regulations, a tobacco control policy innovation, and identified the key attributes of those jurisdictions that successfully pursued this innovation and those that to date have not. In doing so, we employed the actor-network theory, a comprehensive framework for the analysis of fundamental system change. Through our analysis, we identified approaches to help overcome some systemic barriers to the solution of the tobacco problem and comment on other complex transnational problems.	Young D, Borland R, Coghill K. An actor-network theory analysis of policy innovation for smoke-free places: understanding change in complex systems. American Journal of Public Health. 2010;100(7):1208-1217. doi: 10.2105/AJPH.2009.184705

© 2019 Byron M.J.
